# Presentation and evaluation of a modern course in disaster medicine and humanitarian assistance for medical students

**DOI:** 10.1186/s12909-021-03043-6

**Published:** 2021-12-10

**Authors:** Sabine Hermann, Jessica Gerstner, Florian Weiss, Simon Aichele, Eric Stricker, Eleonora Gorgati, Peter Rosenberger, Robert Wunderlich

**Affiliations:** 1 University Department of Anesthesiology and Intensive Care Medicine of Tübingen, Hoppe- Seyler- Straße 3, 72076 Tübingen, Germany; 2 Psychological Institute, Department of Biological and Personality Psychology, University of Freiburg, Freiburg, Germany

**Keywords:** Disaster medicine, Humanitarian assistance, Undergraduate medical education, Germany, Curriculum, Crisis management, Survey/ questionnaires, Medical students, Disaster preparedness curriculum

## Abstract

**Background:**

Disaster medicine is a component of the German medical education since 2003. Nevertheless, studies have shown some inconsistencies within the implementation of the national curriculum, and limits in the number of students trained over the years. Recently, the SARS-CoV-2 pandemic and other disasters have called attention to the importance of training medical students in disaster medicine on a coordinated basis. The aim of this study is to present and evaluate the disaster medicine and humanitarian assistance course, which was developed in the University of Tübingen, Germany.

**Methods:**

The University Clinic for Anesthesiology and Intensive Care Medicine in Tübingen expanded the existing curriculum of undergraduate disaster medicine training with fundamentals of humanitarian medicine, integrating distance learning, interactive teaching and simulation sessions in a 40 h course for third-, fourth- and fifth- year medical students.

This prospective and cross-sectional study evaluates the *Disaster Medicine and Humanitarian Assistance* course carried out over five semesters during the period between 2018 and 2020. Three survey tools were used to assess participants’ previous experiences and interest in the field of disaster medicine, to compare the subjective and objective level of knowledge before and after training, and to evaluate the course quality.

**Results:**

The total number of medical students attending the five courses was *n* = 102 of which *n* = 60 females (59%) and *n* = 42 males (41%). One hundred two students entered the mandatory knowledge assessment, with the rate of correct answers passing from 73.27% in the pre-test to 95.23% in the post-test (*t* [101] = 18.939, *p* < .001, *d* = 1.88). To determine the subjective perception of knowledge data were collected from 107 observations. Twenty-five did not complete the both questionnaires. Out of a remaining sample of 82 observations, the subjective perception of knowledge increased after the course (*t* [81] = 24.426, *p* < .001, *d* = 2.69), alongside with the interest in engaging in the field of disaster medicine (*t* [81] = 7.031, *p* < .001, *d* = .78). The 93.46% of the medical students (*n* = 100) graded the training received with an excellent overall score (1.01 out of 6).

**Conclusion:**

The study indicates a significant increase in students’ understanding of disaster medicine using both subjective and objective measurements, as well as an increase interest in the field of disaster medicine and humanitarian assistance. Whereas former studies showed insufficient objective knowledge regarding disaster medical practices as well as subjective insecurities about their skills and knowledge to deal with disaster scenarios, the presented course seems to overcome these deficiencies preparing future physicians with the fundamentals of analysis and response to disasters. The development and successful implementation of this course is a first step towards fulfilling disaster medicine education requirements, appearing to address the deficiencies documented in previous studies. A possible adaptation with virtual reality approaches could expand access to a larger audience. Further effort must be made to develop also international training programs, which should be a mandatory component of medical schools’ curricula.

**Supplementary Information:**

The online version contains supplementary material available at 10.1186/s12909-021-03043-6

## Background

Disaster medicine is becoming a more recognizable field. The increasing number and intensity of disasters occurring globally are underlining its importance and necessity [1, 2]. The effects of global warming, overpopulation and industrialization, on both national and international scale, will require a future generation of healthcare professional capable of practicing in austere and resource-constrained settings [3, 4]. The current Coronavirus 19- pandemic and the flooding in Western Germany are clear examples. The rise of this global emergency caused an exceptional strain on our healthcare systems, emphasizing the need of educational programs to train future professionals to be able to assist in the disaster response [5].

As of 2003, disaster medicine became a component of German medical schools final examinations [6]. In 2004 the “World Association for Disaster and Emergency Medicine (WADEM; Madison, Wisconsin USA)” pointed out the need of standards and guidelines for the education and training of all the professions involved in disaster response [7]. Although further details like skills, competences or personal attributes were never defined. Consequently in 2006 the German Ministry of Interior (Berlin, Germany), the German Society of Disaster Medicine (Kirchseeon, Germany) and the German Federal Office of Civil Protection and Disaster Assistance (Bonn, Germany) reacted on these recommendations by releasing a curriculum for disaster medicine education at German universities [8]. Four years later in 2010, a 28 h course consisting of 14 key modules was published by Pfenninger at al [9].. Despite that, a nation-wide study published in 2017 showed that the number of trained students and the educational level in the field of disaster medicine in Germany were still low. Moreover, the curriculum of 2006 was not implemented as planned [10]. From these startling findings, in 2018 the University Clinic for Anesthesiology and Intensive Care Medicine in Tübingen developed a 40 h course for students in their third to fifth year of medical school, based upon the 14 key modules of the existing German curriculum for disaster medicine [9] and the experiences of other national courses from Italy, Germany, Saudi Arabia and the International Federation of Medical Students’ Associations IFMSA [4, 8–14]. The theory and practice of international disaster medicine in humanitarian assistance was added to the curriculum according the Humanitarian Charter and Minimum Standards in Humanitarian Response by the Sphere Project*,* a group of humanitarian non-governmental-organizations and the Red Cross and Red Crescent movement originated in the 1990s after Ruanda genocide and recognized as a global model in this field [15].

Students were taught how to maximize the number of casualties saved within limited resource settings through a variety of interactive teaching and learning methods. The main learning objectives were the ability to explain the theoretical basis of national and international disaster medicine and major emergencies, the knowledge and ability to apply preclinical and clinical care as well as the strategies for triage in the event of a disaster. Lastly the familiarity in decontamination concepts in chemical, biological, radioactive, and nuclear emergencies and the ability to apply ethical and psychological considerations in the event of a disaster.

At the beginning, students had to pass a 10 units e-learning course based on the international standards for humanitarian assistance lined out in *The Sphere Handbook in Action* [16], before progressing to the residential part of the course. The residential part consisted of 30 units in 1 week. This comprised a written pre-test, a written post-test, a feedback session and 14 theoretical and 13 practical teaching units. A get-together for the course participants and the presentation of disaster medicine and humanitarian assistance films were organized as optional group activities. A schedule of the complete residential part is provided in Table 1.

**Table 1:**
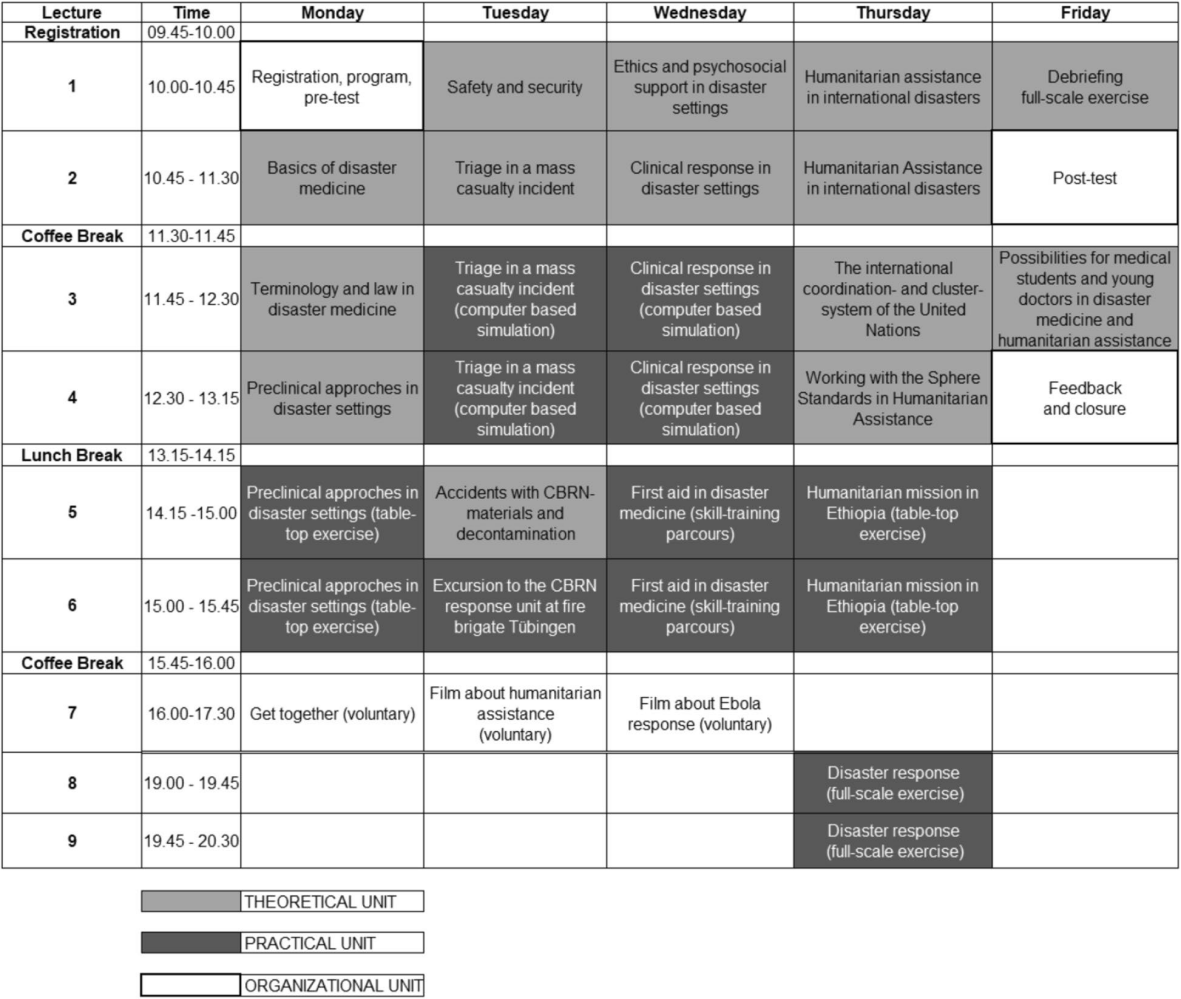
Timetable of the disaster medicine and humanitarian assistance course at the University Department of Anesthesiology and Intensive Care Medicine of Tübingen

To limit the classic frontal lessons and conduct the course in a hands-on approach, modern pedagogic methods including computer-based simulations, tabletop exercises, skills training, role-plays, and a final full-scale live simulation were used. Specifically, the final practical triage exercise was designed as a live simulation to enable medical students applying their theoretical and practical knowledge in an appropriate context, providing a more realistic framework of learning.

The course was delivered in small groups for a close supervision of the learning process providing individualized programs for the students, encouraging immediate feedback with the added benefit of guided practical modules and a deeper learning of topics of special interest. Above the field of disaster medicine, our course addressed the importance of communication and effective teamwork with both known and unknown interdisciplinary staff.

The course was conducted by three experienced medical doctors from the University Department of Anesthesiology and Intensive Care Medicine of Tübingen and supported by the Tübingen Center for Patient Safety and Simulation (TÜPASS), the local emergency services and fire department. The aim of this publication is to present and evaluate the disaster medicine and humanitarian assistance course of the University of Tübingen conducted from 2018 to 2020 according to the requested training for the medical profession by WADEM in 2004.

## Methods

### Study design and setting

This prospective and cross-sectional study presents and evaluates a course in *Disaster Medicine and Humanitarian Assistance* at the University of Tübingen, Germany. The course was conducted every semester for 3 years, between 2018 and 2020 for a total number of five, with the same lecturers as well as topics and structure as illustrated in Table 1. Before and after each course the students were examined with questionnaires on their subjective and objective knowledge in disaster medicine and humanitarian assistance according to the 14 key modules of the German curriculum [9] and the international humanitarian assistance standards [15]. Additionally, the students had to evaluate the course using the evaluation tool of the medical faculty at the end of the lectures.

### Study population

The study population was composed of medical students at their third, fourth and fifth year. The participation in the courses was voluntary and the courses open to 25 students. In the first semester of 2018, 19 students participated. In the second and in the third semester 17 students attended. In the fourth semester 24 students and in the last semester, in 2020, 25. This equals 102 students in total.

### Materials

Three survey tools were used to assess the effectiveness of the course and the students’ interest in the field of disaster medicine. Their outcomes were then considered separately.

The first tool, “Subjective Judgment of Knowledge and Interest in Disaster Medicine”, was a voluntary web-based, purpose-designed subjective questionnaire, which was given to the participants at the beginning and at the end of the course to obtain information about their previous experiences, knowledge and personal interest in the topic (*Supplementary material: Self-Assessment Questionnaire*). Using the same form at two different times allowed comparing knowledge levels before and after attending the lessons. The questionnaire consisted of 26 items. Firstly, the students were asked to choose a pseudonym for both tests. Questions two and three collected demographic data, followed by five yes/no questions to assess the previous experience in the disaster medicine and humanitarian assistance field and 14 to measure the self-reported level of knowledge according to the topics relevant in the curriculum. Additionally, four questions assessed the personal interest in engaging in the field of disaster medicine. These two last groups of questions used an anchored 5-point Likert-Scale (1 = “strongly disagree”; 2 = “disagree”; 3 = “neutral”; 4 = “agree” and 5 = “strongly agree”). The data of the questionnaire were collected with the software SurveyMonkey®, Version 2.0 (SurveyMonkey Europe; Dublin, Ireland).

The second tool, “Objective Measurement of Knowledge”, was a mandatory knowledge pre- and post-test given on the first and on the last day of the residential part of the course. Both tests were used to obtain an objective measurement of knowledge variation and course efficacy, while the post one was also used to evaluate the students to get a mark to pass the course. The two surveys were designed as multiple-choice tests with a single correct answer for each question and consisted of 20 questions about the theory of disaster medicine and ten patient profiles to be triaged. After matching the pre- and post-test results, the number and percentage of correct answers out of 30 were counted and compared. Next, the students’ names were removed and replaced by consecutive numbers for blinded statistical analysis.

The third tool, “Evaluation of the Medical Students’ Satisfaction with the Course”, consisted of the anonymous evaluation system of the University of Tübingen, tuevalon (Ostrakon Software GmbH; Tübingen, Germany), which is used to evaluate all medical students’ courses in Tübingen. The survey contained ten items evaluating the content of the course and six questions regarding satisfaction with the lecturer’s performance. In this study we only analyzed one item of this tool, the overall rating, which was assessed using a 6-point Likert scale according to the German university grading system (1 = “very good”; 2 = “good”; 3 = “satisfactory”; 4 = “sufficient”; 5 = “poor”; 6 = “insufficient”).

The participation to all the three surveys was anonymous and confidential. The participants signed an informed consent form for participation and publication of the assessed data during the course. Additionally, the completion of the questionnaires implied participants’ consent giving authors the right of use of the information provided.

### Statistical analysis

The collected data from the first tool were exported from SurveyMonkey® to Microsoft Excel®, Version 2019 (Microsoft Corporation; Redmond, Washington USA). The analysis was performed with the dedicated statistics program IBM SPSS, Version 22 (IBM Deutschland GmbH; Ehningen, Germany). The overall pre- and post-test scores were obtained by calculating the mean values of the 14 knowledge and 4 personal interest-related items. Paired sample t-tests were conducted for analysis of differences between the overall scores before and after the course regarding medical students’ knowledge and interest. The paired sample t-test was used as first choice statistical test considering its robustness, despite slight violation of the normality assumption within the given data.

The results from the second tool were collected with Microsoft Excel®, Version 2019 and exported to the statistics program IBM SPSS, Version 22 for further analyses. Again, a paired sample t-test was conducted to compare the percentage of correct answers from the mandatory pre- and post-test of knowledge.

Given the lack of systematic evaluations of such courses, it was not possible to conduct an a priori power analysis to determine the necessary sample sizes, neither for the first nor for the second tool. Instead, Cohen’s *d* for paired sample t-tests post-hoc was calculated.

Regarding the third tool, the mean overall rating result was exported from the evaluation system of the University of Tübingen, tuevalon.

## Results

### Demographics

The total number of medical students attending the five courses was *n* = 102 of which *n* = 60 females (59%) and *n* = 42 males (41%), at their third, fourth and fifth year of medical school.

### Subjective judgment of knowledge and interest in disaster medicine

*N* = 107 observations were collected prior to the course. Participants were included from semester six to ten with a median of semester eight. Since *n* = 22 participants did not complete the questionnaire after the course and the pseudonyms of *n* = 3 participants at the post-test could not be assigned to the corresponding pre-test data, the final sample size comprised *n* = 82 for statistical analysis (Table 2).

**Table 2:** Subjective Judgment of Knowledge and Interest in Disaster Medicine

Results		Pre	Post	Paired sample t-test
First tool (*n* = 82)	A. Subjective judgement of knowledge	*m* = 2.37 *SD* = .74	*m* = 4.24 *SD* = .35	*t*(81) 24.426, *p* < .001, *d* = 0.78
	B. Subjective judgement of interest	*m* = 3.85 *SD* = .54	*m* = 4.21 *SD* = .49	*t*(81) = 7.031, *p* < .001, *d* = 2.69

Within this sample, the medical students had no previous experience (*m* = 1.29, *SD* = .02) with answers ranging from “strongly disagree” = 1 to “disagree” = 2. Statistical significant differences (*t* [81] = 24.426, *p* < .001, *d* = 2.69) were identified in the participants’ subjective perception of knowledge before and after the course.

**Fig. 1:**
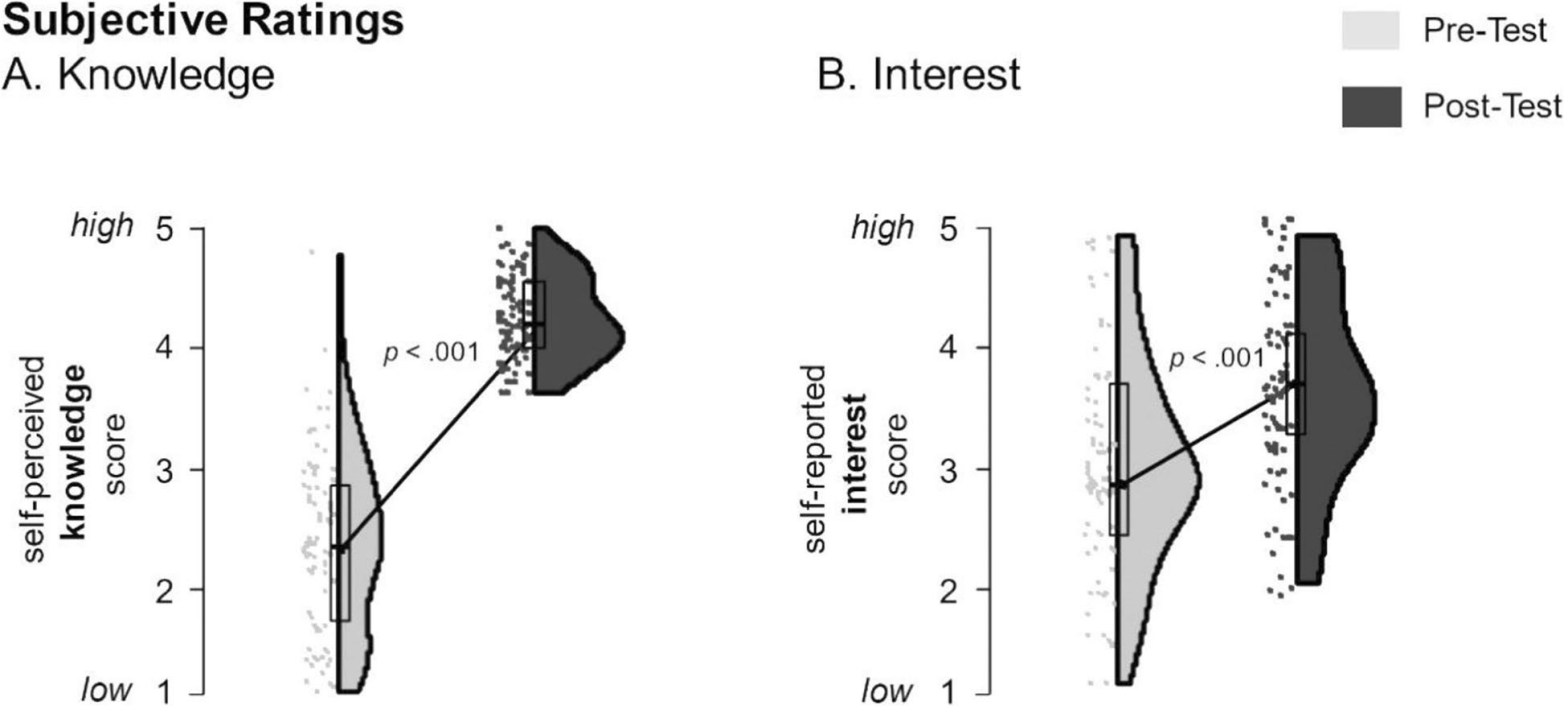
Self-perception of knowledge ratings (**A**) and interest (**B**) in disaster medicine and humanitarian assistance pre-course and post-course

Likewise, students’ future interest in engaging in the field of disaster medicine after the course showed a statistically significant increase (*t* [81] = 7.031, *p* < .001, *d* = .78), although medical students’ interest was already high prior to the course (*m* = 3.85, *SD* = .54), which could explain the smaller effect size (Fig. 1).

### Objective measurement of knowledge

**Fig. 2:**
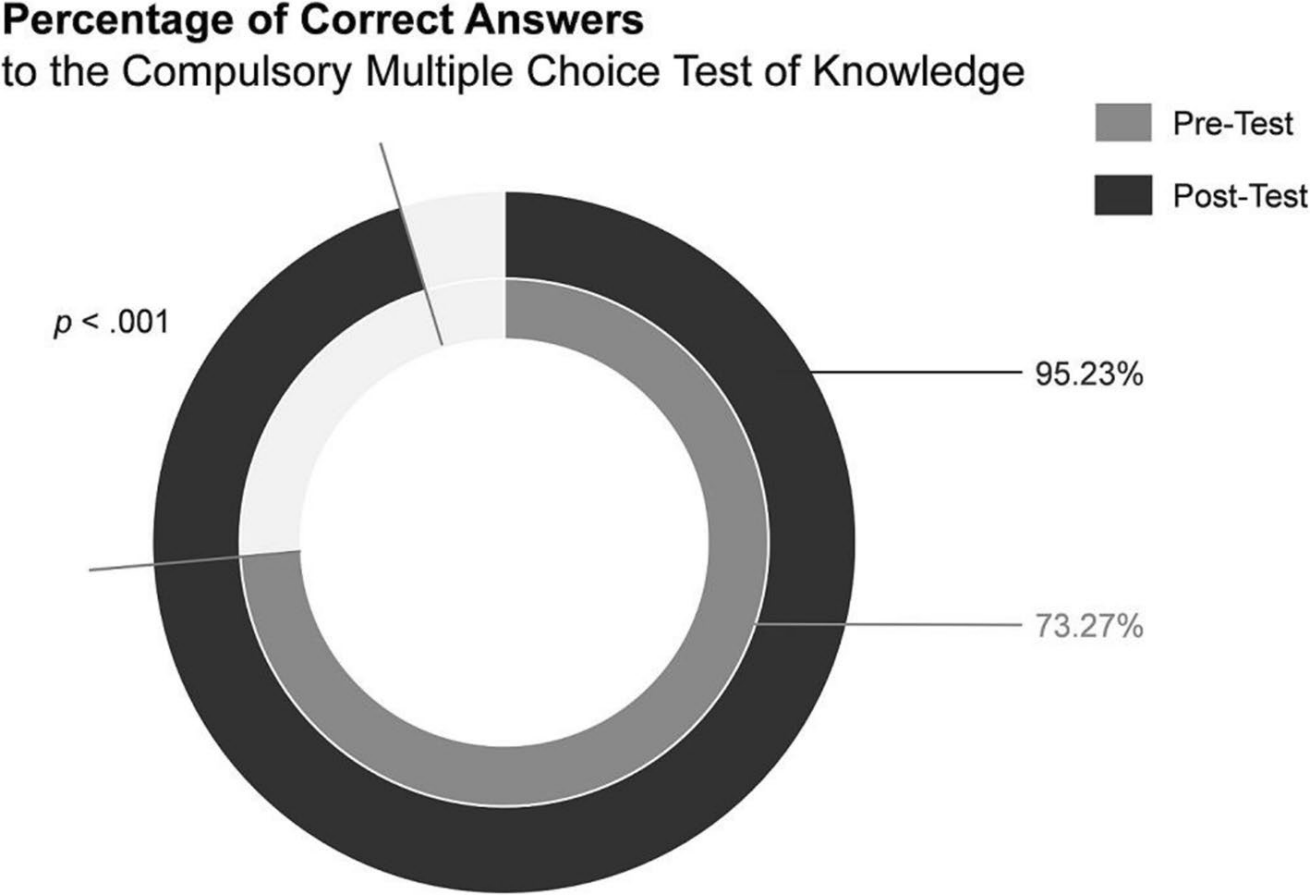
Correct answers to the multiple-choice test of knowledge in disaster medicine and humanitarian assistance

A total number of *n* = 102 medical students entered the compulsory multiple-choice pre- and post- test of knowledge. Participants’ rate of correct answers changed from 73.27% (*SD* = 11.34%) in the pre-test to 95.23% (*SD* = 4.75%) in the post-test (Fig. 2*)*, with this difference being statistically significant (*t* [101] = 18.939, *p* < .001, *d* = 1.88) *(*Table 3).

**Table 3:** Objective Measurement of Knowledge

Results		Pre	Post	Paired sample t-test
Second tool (*n* = 102)	Percentage of correct answers to the mandatory test of knowledge	*m* = 73.27% *SD* = 11.34%	*m* = 95.23% *SD* = 4.75%	*t* (101) = 18.939, *p* < .001, *d* = 1.88

### Evaluation of the medical students’ satisfaction with the course

Regarding the evaluation system of the University of Tübingen tuevalon, qualitative data of 98.04% (*n* = 100) of the medical students who participated between 2017 and 2020 were analyzed. The course took place on five separate occasions and received an overall grade of 1.01 (1 = “very good”) as rated by participants.

## Discussion

Natural and human-caused disasters are increasingly threatening the international community, and a severe lack in adequate disaster medicine education has been identified [10]. In consequence, the *Disaster Medicine and Humanitarian Assistance* course of the University of Tübingen, Germany, was established as an appropriate response to the “call-to-action” promoting and enhancing training capacity in the field of disaster medicine.

This prospective and cross-sectional study provides a presentation and evaluation of the mentioned new course for medical students at their third to fifth year at the University of Tübingen, conducted over five semesters between 2018 and 2020.

Three survey tools were used to assess the effectiveness of the course in terms of students’ knowledge and interest in the field of disaster medicine before and after their participation to the course. Additionally, the students’ satisfaction with the course was measured. The results indicate a significant increase in students’ understanding and knowledge of disaster medicine and humanitarian assistance, in both subjective and objective measurements, showing higher scores after the course experience. Furthermore, the statistical analyses stress an increase in students’ future interest in the field of disaster medicine upon completion of the course including their overall satisfaction regarding the implementation of the training. The practical core of the course and the focus on simulation formats might have played a significant role.

Whereas former studies showed insufficient objective knowledge regarding disaster medical practices [10] as well as subjective insecurities about their skills and knowledge to deal with disaster scenarios [17], the presented course appears to overcome these deficiencies. Further, it seems to fulfil the desires of medical students for a comprehensive and practically relevant course on disaster medicine and its inclusion as a pillar of academic curricula. While in the most recent programs great heterogeneity is observed - the Italian *Research Center in Emergency and Disaster Medicine (CRIMEDIM)* focuses purely on theoretical lectures [12] and some American approaches particularly address terror scenarios [18, 19] - the presented Disaster Medicine and Humanitarian Assistance course comprehensively combines a multitude of teaching methods, including the use of disaster scenarios and involving ethical and psychological aspects, all elements which might have contribute to increase the students’ interest and effective impression of the training experience.

### Limitations

Although we assessed the increase in knowledge through pre- and post-measurement of objective and subjective learning, we did not evaluate the acquired practical skills in detail.

We cannot exclude participation bias within our data, as not all medical students completed the post-test regarding their subjective judgement of knowledge. Therefore, the collected data might be restricted to those who felt confident about their gained knowledge or who were satisfied with the course – hence, careful interpretation of these results is required. A noteworthy aspect is that medical students’ interest in working in the field of disaster medicine was already high prior to course attendance. This also reflected in the pre-test’s results, where the mean percentage of correct answers was already around 75%, showing a preliminary knowledge on the topics covered in the course.

## Conclusion

The importance of a *Disaster Medicine and Humanitarian Assistance* course is highlighted now, as the spread of the SARS-CoV-2 pandemic is threatening the international community and pushing medical care systems to their limits [20]. With its multi-method, comprehensive, well-developed foundation and practical relevance, the presented course of the University of Tübingen provides an appropriate response to the urgent need for effective disaster-medicine training programs, preparing future physicians with the fundamentals to understand and respond to disasters. Further effort must be made to develop also international training programs and promote international and interdisciplinary exchanges for disaster medicine, which should become a mandatory component of medical schools’ curricula. Future studies should hence include an objective measurement of the medical students’ practical performance, which for example could be attained with computer or full-scale simulations. Furthermore, the comparison of a broader range of approaches could give an insight in the efficacy of different disaster medicine training programs in general, and different teaching methods. Since the practical modules of this course, especially the final full-scale simulation, require many resources in terms of structures, materials and staff, virtual reality (VR) adaptations could be considered as an alternative to implement the course more quickly to a larger number of medical schools on a national and international level [21]. Further studies could focus on the implementation and effectiveness of VR-adaptations in disaster medicine courses.

## Supplementary Information

**Additional file 1.**

**Additional file 2.**

**Additional file 3.**

**Additional file 4.**

**Additional file 5.**

## Data Availability

The datasets used and/or analyzed during the current study are available from the corresponding author on reasonable request.

## Abbreviations

CRIMEDIM: Research Center in Emergency and Disaster Medicine
VR: Virtual Reality
WADEM: World Association for Disaster and Emergency Medicine
TÜPASS: Tübingen Center for Patient Safety and Simulation
IFMSA: International Federation of Medical Students’ Associations
