# Supplementary material for: Presentation and evaluation of a modern course in disaster medicine and humanitarian assistance for medical students
Source: BMC Med Educ. 2021 Dec 10;21:610. doi: 10.1186/s12909-021-03043-6 (PMC8661312; doi:10.1186/s12909-021-03043-6)
Supplement: Supplementary file 1 — Additional file 1. [file 12909_2021_3043_MOESM1_ESM.pdf]

TÜKLIS Katastrophenmedizin und Humanitäre Hilfe

Einführung / Introduction

Mit der Teilnahme am Fragebogen stimmen Sie mit wissenschaftlichen Auswertung und Veröffentlichung der erhobenen Daten zu. Sollten Sie dies nicht wünschen nehmen Sie bitte nicht am Fragebogen teil und wenden sich an die Kursleitung.

1. Bitte legen Sie ein Pseudonym für sich fest, welches Sie beim ersten und zweiten Ausfüllen der Datenerhebung nutzen können!

Please use a pseudonym you can use for both times of answering the questionnaire!

* 2. Geschlecht

Sex

- Weiblich / Female
- Männlich / Male

* 3. In welchem Fachsemester studieren Sie ?

In which regular semester do you study?

TÜKLIS Katastrophenmedizin und Humanitäre Hilfe

Allgemeine Fragen zur Katastrophenmedizin / General questions about disaster medicine

Bitte beantworten Sie nun die Fragen zu Ihrem persönlichen Vorwissen / Please answer now some questions about your previous knowledge

* 4. Haben Sie früher bereits vom Fach "Katastrophenmedizin und Humanitäre Hilfe" gehört?

Did you hear about the subject „disaster medicine“ before?

- Nein / No
- Ja / Yes

* 5. Haben Sie eine abgeschlossene Berufsausbildung im Rettungsdienst oder der Humanitären Hilfe?

Did you finish a higher education in rescue services?

- Nein / No
- Ja / Yes

6. Sind Sie bereits ehrenamtlich in einer Hilfsorganisation tätig?

Are you already voluntary working in an aid organization?

- Nein / No
- Ja / Yes

* 7. Haben Sie in Ihrer jetzigen Universität Unterricht zum Thema „Katastrophenmedizin und Humanitäre Hilfe“ erhalten?

Did you have lectures about „disaster medicine“ in your actual medical school?

- Nein / No
- Ja / Yes

* 8. Haben Sie in einer anderen Einrichtung Unterricht in Katastrophenmedizin erhalten?

Did you have lectures in disaster medicine somewhere else?

- Nein / No
- Ja / Yes

Fragen angeleht an das Curriculum / Questions according to the curriculum

Bitte schätzen Sie selbst Ihr Wissen zur Katastrophenmedizin und Humanitären Hilfe ein /Please assess your knowledge of disaster medicine

* 9. Ich kenne die Terminologie der Katastrophenmedizin, die rechtlichen Aspekte, sowie die Katastrophentypen
I know the terminology of disaster medicine, legal aspects, as well as the disaster classification

Ich stimme gar nicht zu<br>I strongly disagree	Ich stimme nicht zu<br>I disagree	neutral<br>neutral	Ich stimme zu<br>I agree	Ich stimme entschieden zu<br>I strongly agree
◯	◯	◯	◯	◯

* 10. Ich kenne die Organisation und Führungslehre, um einen Massenanfall an Verletzten zu bewältigen
I know the organization and leadership to deal with a large number of casualties

Ich stimme garnicht zu<br>I strongly disagree	Ich stimme nicht zu<br>I disagree	neutral<br>neutral	Ich stimme zu<br>I agree	Ich stimme entschieden zu<br>I strongly agree
◯	◯	◯	◯	◯

* 11. Ich weiß wie man mit eine große Anzahl an Verletzten, die die normale Kapazität des medizinischen Systems überschreiten, medizinisch versorgen muss
I know how to deal with a number of patients, which is exeeding the normal capacity of the medical system

Ich stimme garnicht zu<br>I strongly disagree	Ich stimme nicht zu<br>I disagree	neutral<br>neutral	Ich stimme zu<br>I agree	Ich stimme entschieden zu<br>I strongly agree
◯	◯	◯	◯	◯

* 12. Ich weiß wie die Alarmierung und Evakuierung einer Klinik, im Fall einer externen Katastrophe, abläuft

I know how the basics about alarm and evacuation of hospitals in case of an external disaster

Ich stimme gar nicht zu<br>I strongly disagree	Ich stimme nicht zu<br>I disagree	neutral<br>neutral	Ich stimme zu<br>I agree	Ich stimme entschieden zu<br>I strongly agree
○	○	○	○	○

* 13. Ich kann die Umsetzbarkeit von medizinischer Katastrophenhilfe in Einsatzbeispielen beurteilen

I can evaluate the practicability of medical disaster response in practical examples

Ich stimme gar nicht zu<br>I strongly disagree	Ich stimme nicht zu<br>I disagree	neutral<br>neutral	Ich stimme zu<br>I agree	Ich stimme entschieden zu<br>I strongly agree
○	○	○	○	○

* 14. Ich kann die Prinzipien der Sichtung im präklinischen und klinischen Setting anwenden

I can use the principles of Triage in a preclinical and clinical setting

Ich stimme gar nicht zu<br>I strongly disagree	Ich stimme nicht zu<br>I disagree	neutral<br>neutral	Ich stimme zu<br>I agree	Ich stimme entschieden zu<br>I strongly agree
○	○	○	○	○

TÜKLIS Katastrophenmedizin und Humanitäre Hilfe

Fragen angeleht an das Curriculum / Questions according to the curriculum

Bitte schätzen Sie selbst Ihr Wissen zur Katastrophenmedizin ein/ Please assess your knowledge in disaster medicine

* 15. Ich kenne die Grundlagen der basismedizinischen Versorgung unter Katastrophenbedingungen (lebensrettende Sofortmaßnahmen)
I know the basics of primary health care under disaster circumstances (life-saving procedures)

Ich stimme gar nicht zu<br>I strongly disagree	Ich stimme nicht zu<br>I disagree	neutral<br>neutral	Ich stimme zu<br>I agree	Ich stimme entschieden zu<br>I strongly agree
◯	◯	◯	◯	◯

* 16. Mir sind die Grundlagen der medizinischen Versorgung bei Unfällen mit radioaktiven Materialien, sowie Dekontaminationsmaßnahmen, bekannt.
I am aware of the medicial management after accidents with radioactive materials and the decontamination of these.

Ich stimme gar nicht zu<br>I strongly disagree	Ich stimme nicht zu<br>I disagree	neutral<br>neutral	Ich stimme zu<br>I agree	Ich stimme entschieden zu<br>I strongly agree
◯	◯	◯	◯	◯

* 17. Mir sind die Grundlagen des Managements von Gefahrguttransporten und - unfällen, sowie Massenvergiftungen mit Chemikalien sowie Dekontaminationsmaßnahmen, bekannt.
I am aware of the management of transports of hazardos material and accidents as well as the management of mass intoxications with chemicals and decontamination.

Ich stimme gar nicht zu<br>I strongly disagree	Ich stimme nicht zu<br>I disagree	neutral<br>neutral	Ich stimme zu<br>I agree	Ich stimme entschieden zu<br>I strongly agree
◯	◯	◯	◯	◯

* 18. Ich kenne die Grundsätze des ethischen Handelns in der Katastrophenmedizin und das Qualitätsmanagement in der Katastrophenhilfe.

I know the basics of ethical action in disaster medicine and the quality management in disaster response.

Ich stimme gar nicht zu<br>I strongly disagree	Ich stimme nicht zu<br>I disagree	neutral<br>neutral	Ich stimme zu<br>I agree	Ich stimme entschieden zu<br>I strongly agree
◯	◯	◯	◯	◯

* 19. Ich kenne die Grundlagen der durch Katastrophenstress ausgelösten Erkrankungen und die Maßnahmen psychosozialer Unterstützung in Katastrophensituationen.

I know the basics of diseases triggered by disaster situations and the actions of psychosocial support in disaster situations.

Ich stimme gar nicht zu<br>I strongly disagree	Ich stimme nicht zu<br>I disagree	neutral<br>neutral	Ich stimme zu<br>I agree	Ich stimme entschieden zu<br>I strongly agree
◯	◯	◯	◯	◯

* 20. Ich kenne die Grundlagen der Arbeitsweisen in der Humanitären Hilfe

I have the knowledge in the standard working procedures of humanitarian assistance.

Ich stimme gar nicht zu<br>I strongly disagree	Ich stimme nicht zu<br>I disagree	neutral<br>neutral	Ich stimme zu<br>I agree	Ich stimme entschieden zu<br>I strongly agree
◯	◯	◯	◯	◯

* 21. Ich kenne das internationale Koordinierungs- und Clustersystem der Vereinten Nationen

Ich stimme gar nicht zu<br>I strongly disagree	Ich stimme nicht zu<br>I disagree	neutral<br>neutral	Ich stimme zu<br>I agree	Ich stimme entschieden zu<br>I strongly agree
◯	◯	◯	◯	◯

* 22. Ich kann mit den Humanitären Standards des Sphere Handbooks arbeiten

Ich stimme gar nicht zu<br>I strongly disagree	Ich stimme nicht zu<br>I disagree	neutral<br>neutral	Ich stimme zu<br>I agree	Ich stimme entschieden zu<br>I strongly agree
◯	◯	◯	◯	◯

TÜKLIS Katastrophenmedizin und Humanitäre Hilfe

Persönliches Interesse an Katastrophenmedizin / Personal interest for disaster medicine

Bitte beantworten Sie nun die Fragen zu Ihrem persönlichen Interesse an Katastrophenmedizin /
Please answer now some questions about your personal interest in disaster medicine

* 23. Ich bin interessiert daran (weiter) über Katastrophenmedizin und Humanitäre Hilfe zu lernen
I am interested to learn about disaster medicine

Ich stimme gar nicht zu<br>I strongly disagree	Ich stimme nicht zu<br>I disagree	neutral<br>neutral	Ich stimme zu<br>I agree	Ich stimme entschieden zu<br>I strongly agree
◯	◯	◯	◯	◯

* 24. Es ist notwendig das Fach "Katastrophenmedizin und Humanitäre Hilfe" im Lehrplan des Medizinstudiums verpflichtend zu verankern.
It is neccessary to put the compulsery subject "disaster medicine" in the curriculum for human medicine

Ich stimme gar nicht zu<br>I strongly disagree	Ich stimme nicht zu<br>I disagree	neutral<br>neutral	Ich stimme zu<br>I agree	Ich stimme entschieden zu<br>I strongly agree
◯	◯	◯	◯	◯

* 25. Ich will in der Zukunft in der Katastrophenmedizin arbeiten

I want to work in the field of disaster medicine in the future

Ich stimme gar nicht zu<br>I strongly disagree	Ich stimme nicht zu<br>I disagree	neutral<br>neutral	Ich stimme zu<br>I agree	Ich stimme entschieden zu<br>I strongly agree
○	○	○	○	○

* 26. Ich will in der Zukunft in der Humanitären Hilfe arbeiten

I want to work in the field of disaster medicine in the future

Ich stimme gar nicht zu<br>I strongly disagree	Ich stimme nicht zu<br>I disagree	neutral<br>neutral	Ich stimme zu<br>I agree	Ich stimme entschieden zu<br>I strongly agree
○	○	○	○	○

TÜKLIS Katastrophenmedizin und Humanitäre Hilfe

Vielen Dank! / Thank you very much!

Vielen Dank für Ihre Teilnahme!

Thank you very much for your participation!
