# Supplementary material for: Presentation and evaluation of a modern course in disaster medicine and humanitarian assistance for medical students
Source: BMC Med Educ. 2021 Dec 10;21:610. doi: 10.1186/s12909-021-03043-6 (PMC8661312; doi:10.1186/s12909-021-03043-6)
Supplement: Supplementary file 2 — Additional file 2. [file 12909_2021_3043_MOESM2_ESM.pdf]

Supplemental Material: Raw Data Subjective Judgment of Knowledge and Interest in Disaster Medicine

code	dropout	measuringpoint	sex	semester	preexperience_item_1	preexperience_item_2	preexperience_item_3	preexperience_item_4	preexperience_item_5	knowledge_item_1	knowledge_item_2	knowledge_item_3	knowledge_item_4	knowledge_item_5	knowledge_item_6	knowledge_item_7	knowledge_item_8	knowledge_item_9	knowledge_item_10	knowledge_item_11	knowledge_item_12	knowledge_item_13	knowledge_item_14	interest_item_1	interest_item_2	interest_item_3	interest_item_4
1	0	1	2	9	2	2	1	1	2	3	3	2	1	2	3	4	1	1	3	2	2	2	3	5	5	4	4
2	0	1	2	7	2	1	1	1	1	2	1	2	2	3	3	3	2	2	3	3	3	1	4	4	3	3	3
3	0	1	1	8	2	1	2	1	2	2	2	2	2	2	2	4	2	2	4	2	3	2	2	5	5	5	5
4	0	1	1	8	2	2	1	1	2	2	4	4	2	2	4	4	1	2	4	4	2	1	2	5	5	4	2
5	0	1	2	7	1	1	1	1	1	2	1	2	2	2	2	2	1	1	1	3	1	1	3	4	3	3	2
6	0	1	2	7	2	2	2	1	2	3	5	5	2	4	5	5	4	4	4	4	2	2	2	4	4	4	3
7	0	1	2	6	1	2	2	1	1	2	3	3	2	2	2	4	2	1	1	4	3	1	3	4	3	3	3
8	0	1	2	9	2	1	1	1	1	2	2	2	1	2	1	2	1	1	1	2	1	1	1	5	3	4	3
9	0	1	2	6	1	2	2	1	2	3	4	4	2	2	4	4	2	2	1	2	3	2	3	5	3	4	2
10	0	1	1	8	2	2	1	1	2	3	4	4	2	3	4	4	2	2	4	4	4	3	4	5	4	4	4
11	0	1	2	8	2	2	1	1	2	2	3	4	2	2	3	4	1	2	2	3	3	1	1	5	5	5	5
12	1	1	2	8	1	2	1	1	2	2	3	2	1	1	3	4	1	1	2	2	3	1	1	5	5	4	4
13	0	1	1	8	1	1	2	1	1	1	1	1	1	1	2	4	1	1	2	3	2	2	1	5	4	3	3
14	1	1	2	6	1	1	1	1	1	1	2	2	1	2	1	3	2	2	2	3	2	1	3	5	4	3	3
15	0	1	1	7	2	1	1	1	1	1	1	1	1	1	1	3	1	1	1	2	1	1	1	4	5	3	3
16	0	1	1	7	2	1	2	1	2	2	4	3	1	1	5	4	1	1	1	1	3	1	1	5	3	3	5
17	1	1	1	10	1	1	2	1	1	2	2	2	1	1	1	4	1	1	1	1	1	1	1	5	5	3	3
18	1	1	2	6	1	2	2	1	1	2	3	2	1	2	3	4	2	2	1	3	1	1	1	5	3	4	3
19	0	1	2	6	2	2	2	1	1	2	4	2	2	2	3	3	3	3	1	2	1	1	1	5	2	4	4
20	1	1	2	7	2	2	2	1	2	4	4	4	3	4	4	5	3	3	4	4	4	2	2	4	4	3	3
21	0	1	1	10	1	1	1	1	1	3	2	2	3	2	3	4	2	2	3	3	2	2	4	5	3	4	4
22	0	1	2	9	2	2	1	1	2	3	4	4	4	3	4	4	3	3	3	3	3	2	3	4	4	4	3
23	0	1	2	10	2	1	2	1	2	3	3	2	2	3	4	4	1	3	4	4	4	2	4	5	4	4	4
24	0	1	1	9	1	1	2	1	1	2	1	1	1	1	2	3	3	2	3	2	2	3	2	4	3	2	4
25	0	1	1	7	2	1	1	1	1	1	1	1	1	1	1	4	1	1	2	2	3	3	3	4	3	2	3
26	0	1	2	8	1	1	2	1	2	2	2	2	2	2	2	4	1	1	2	2	3	1	4	4	3	4	4
27	0	1	2	8	2	2	1	1	2	3	3	3	2	2	4	5	3	2	2	2	1	2	3	4	3	3	2
28	0	1	1	9	2	1	1	1	1	4	2	2	2	2	3	4	2	2	4	4	3	1	4	5	4	4	5
29	0	1	2	8	2	1	1	1	1	1	1	1	2	1	2	2	1	1	1	2	1	1	3	4	4	3	3
30	0	1	2	5	1	1	1	1	1	2	2	2	2	2	2	3	2	2	3	3	2	2	3	5	4	3	3
31	1	1	2	9	2	1	1	1	1	1	1	1	1	1	1	1	1	1	1	1	1	1	1	5	3	3	3
32	0	1	2	8	1	1	1	1	2	3	4	3	3	3	4	4	3	3	4	4	3	2	4	4	3	3	3
33	0	1	1	7	1	1	1	1	1	1	1	1	1	3	1	3	1	1	4	4	4	3	4	5	5	4	4
34	0	1	2	9	2	2	2	1	1	3	5	5	4	4	5	5	4	4	3	3	4	3	4	5	5	4	5
35	0	1	1	6	2	1	1	1	1	3	2	2	2	3	2	2	1	1	4	3	4	3	4	5	4	3	3
36	0	1	2	8	1	1	1	1	1	1	3	2	1	2	2	3	1	1	1	3	1	1	1	5	3	2	4
37	1	1	2	7	1	1	1	1	1	2	2	1	1	2	2	4	2	2	2	1	2	1	2	3	3	2	2
38	0	1	2	8	1	1	1	1	1	2	1	1	1	1	1	2	1	1	1	1	3	2	3	4	3	3	3
39	1	1	1	7	2	1	1	1	1	1	1	2	1	1	1	2	1	1	2	1	1	1	3	5	3	3	3
40	0	1	1	10	2	2	2	1	2	3	4	4	2	3	3	4	2	2	2	3	3	1	3	5	5	4	4
41	0	1	1	7	2	1	1	1	1	1	1	2	1	1	1	4	1	1	3	2	2	1	4	5	3	3	3
42	0	1	2	7	2	1	1	1	1	2	2	2	2	2	2	3	2	1	3	3	3	1	3	4	4	2	2
43	0	1	2	8	1	2	2	1	1	3	4	3	3	3	4	3	2	2	3	4	3	2	2	3	3	2	3
44	0	1	1	8	2	1	1	1	1	3	4	2	3	3	2	4	4	3	4	3	3	2	4	5	5	5	5
45	0	1	1	8	2	1	1	1	2	3	4	4	2	1	4	3	1	2	3	4	3	1	3	5	4	4	3
46	0	1	1	7	1	1	1	1	1	1	1	1	1	1	1	1	1	1	1	1	1	1	3	5	5	5	5
47	0	1	1	6	2	1	1	1	1	1	3	3	1	2	3	3	1	1	3	2	2	1	4	5	4	4	4
48	0	1	1	7	2	1	1	1	1	1	1	1	2	1	1	1	1	1	1	1	2	1	2	5	4	3	3
49	1	1	1	9	1	1	1	1	1	2	1	1	1	1	1	2	1	1	1	2	3	1	3	4	5	3	3
50	0	1	1	7	2	1	1	1	1	2	1	2	2	2	2	2	1	1	2	2	2	2	2	4	4	4	3
51	1	1	1	8	2	1	1	1	1	2	2	3	2	2	3	4	2	2	3	3	3	2	3	3	3	3	3
52	0	1	2	8	1	1	1	1	1	3	2	4	3	4	2	4	1	1	1	4	2	1	1	4	2	3	3
53	1	1	1	7	1	1	1	1	1	2	2	2	2	2	2	2	2	2	2	2	2	1	2	5	5	4	5
54	0	1	1	8	2	1	1	1	1	2	1	1	2	1	1	3	1	1	3	4	2	1	1	5	4	3	3
55	0	1	1	7	1	1	1	1	1	1	1	1	1	1	1	1	1	1	2	2	2	2	3	5	4	3	3
56	0	1	1	8	2	1	1	1	1	1	1	2	1	1	1	4	1	1	1	2	1	1	1	5	4	3	3
57	0	1	2	10	1	1	1	1	1	3	2	1	1	2	3	3	2	2	4	3	3	3	4	5	3	4	4
58	0	1	2	7	2	2	1	1	2	2	4	4	4	2	4	4	2	3	2	4	2	2	2	5	5	5	4
59	0	1	2	8	2	2	1	2	2	4	4	4	2	4	4	5	4	4	4	3	2	1	4	4	3	4	4
60	1	1	2	8	2	2	2	1	2	1	4	4	2	3	4	5	4	4	3	2	2	1	1	3	3	4	2
61	0	1	2	7	2	2	2	1	1	3	4	4	2	4	4	5	2	2	3	2	3	2	2	4	1	3	3
62	0	1	1	5	1	2	2	1	2	3	4	4	2	2	2	4	1	1	2	1	2	1	1	5	4	4	3
63	0	1	2	8	2	2	2	1	2	5	5	5	5	5	5	5	4	4	5	5	5	4	5	5	5	5	5
64	0	1	2	9	2	1	1	1	1	3	3	3	3	3	3	3	3	3	3	3	3	3	3	5	5	3	4
65	0	1	1	10	2	2	2	1	2	4	4	4	2	4	4	4	4	4	4	4	3	2	2	4	4	3	3
66	1	1	1	9	2	1	1	2	2	3	2	2	2	3	2	3	3	2	3	3	3	2	3	5	4	4	4
67	0	1	1	7	2	2	2	1	1	4	4	4	2	2	4	3	3	2	3	3	4	1	3	4	4	4	5
68	0	1	1	8	1	1	1	1	1	1	1	1	1	1	1	3	1	1	1	1	1	1	2	4	4	3	3
69	0	1	1	7	2	1	1	1	1	2	1	1	2	2	1	1	1	1	1	2	1	1	3	5	4	3	3
70	1	1	1	7	2	1	1	1	1	3	1	1	1	1	1	3	1	2	1	1	1	1	2	5	4	3	3
71	1	1	1	7	1	1	1	1	1	1	1	2	1	1	2	3	3	2	3	4	3	1	3	4	3	3	3
72	0	1	1	7	2	1	2	1	1	4	3	4	2	3	4	4	2	2	4	4	3	3	4	5	5	4	3
73	0	1	1	7	1	1	1	1	1	2	2	2	2	2	3	4	2	2	2	2	2	2	3	5	5	4	4
74	1	1	2	7	2	1	1	1	2	2	2	3	1	2	4	4	1	1	4	3	2	2	3	5	4	4	4
75	0	1	2	7	1	1	1	1	2	4	3	2	1	2	3	3	1	2	2	2	2	1	3	5	5	3	2
76	0	1	2	9	1	2	2	1	2	4	4	4	3	3	4	4	3	4	4	4	4	2	4	5	5	5	4
77	0	1	1	9	1	1	1	1	1	1	1	2	1	2	2	2	1	1	1	3	1	1	2	5	4	3	3
78	0	1	1	7	2	1	1	1	1	1	1	1	1	2	2	2	1	1	2	1	2	1	2	5	4	3	3
79	0	1	2	7	2	2	1	1	1	2	4	3	2	2	3	3	1	2	2	3	2	1	2	4	3	3	3
80	0	1	1	8	2	1	1	1	1	2	1	2	2	2	2	3	2	2	3	3	3	1	3	5	5	3	4
81	0	1	1	9	2	2	2	1	2	4	4	4	2	4	3	4	4	4	3	4	2	1	2	5	5	5	2
82	0	1	1	8	1	1	1	1	1	1	1	1	1	2	1	3	1	1	3	3	1	1	3	5	4	3	3
1	0	2	2	9	2	2	1	1	1	4	4	5	3	4	5	5	5	4	5	3	5	5	5	5	5	5	5
2	0	2	2	7	2	1	1	1	1	4	4	5	4	4	5	4	4	4	4	4	5	4	4	4	4	3	4
3	0	2	1	8	2	1	1	1	2	4	4	4	3	4	4	5	4	4	4	4	4	4	4	5	5	5	4
4	0	2	1	8	1	2	1	1	2	4	5	5	2	4	5	5	4	3	4	4	5	4	4	5	5	4	4
5	0	2	2	7	2	1	1	2	1	5	4	5	3	4	5	5	4	2	4	3	3	3	4	4	4	3	2
6	0	2	2	7	2	2	2	1	2	5	5	5	5	5	5	5	5	5	5	5	5	5	5	5	5	5	4
7	0	2	2	6	2	2	1	1	1	5	5	4	4	5	5	5	5	5	5	5	5	5	5	5	4	4	3
8	0	2	2	9	1	1	1	2	1	4	5	5	4	5	5	5	5	5	5	4	4	4	4	4	3	5	2
9	0	2	2	6	1	2	2	2	2	5	4	5	4	5	5	5	4	5	5	5	4	5	5	5	3	5	4
10	0	2	1	8	1	2	1	1	2	4	4	4	2	4	5	4	4	4	4	2	4	4	4	4	4	4	4
11	0	2	2	8	2	2	1	1	2	4	4	5	5	5	5	5	4	3	5	5	5	4	4	5	5	4	5
12	1	2	2	8	-99	-99	-99	-99	-99	-99	-99	-99	-99	-99	-99	-99	-99	-99	-99	-99	-99	-99	-99	-99	-99	-99	-99
13	0	2	1	8	1	1	2	1	1	4	5	5	4	4	5	5	4	4	4	5	4	4	4	5	5	2	4
14	1	2	2	6	-99	-99	-99	-99	-99	-99	-99	-99	-99	-99	-99	-99	-99	-99	-99	-99	-99	-99	-99	-99	-99	-99	-99
15	0	2	1	7	1	1	1	1	1	4	4	4	3	4	5	4	3	3	5	5	5	3	4	4	5	3	3
16	0	2	1	7	2	1	2	1	2	4	4	5	4	5	5	5	4	4	5	5	5	4	5	5	3	4	4
17	1	2	1	10	-99	-99	-99	-99	-99	-99	-99	-99	-99	-99	-99	-99	-99	-99	-99	-99	-99	-99	-99	-99	-99	-99	-99
18	1	2	2	6	-99	-99	-99	-99	-99	-99	-99	-99	-99	-99	-99	-99	-99	-99	-99	-99	-99	-99	-99	-99	-99	-99	-99
19	0	2	2	6	2	2	2	1	1	4	5	4	5	4	5	5	4	4	4	5	5	5	4	5	4	5	4
20	1	2	2	7	-99	-99	-99	-99	-99	-99	-99	-99	-99	-99	-99	-99	-99	-99	-99	-99	-99	-99	-99	-99	-99	-99	-99
21	0	2	1	10	1	1	1	1	1	5	5	5	4	5	5	4	4	4	5	4	5	5	4	5	5	4	4
22	0	2	2	9	2	2	1	1	2	5	5	5	4	4	5	5	4	5	5	4	5	5	5	5	4	4	4
23	0	2	2	10	2	2	2	2	2	4	5	5	4	4	5	5	4	4	4	4	5	5	5	5	5	5	5
24	0	2	1	9	2	1	2	1	1	4	4	4	3	3	4	4	4	3	4	4	4	3	3	4	3	3	4
25	0	2	1	7	1	1	1	1	1	4	5	5	4	4	5	5	5	4	4	5	4	4	4	4	5	2	4
26	0	2	2	8	1	1	2	1	1	4	4	4	3	5	5	5	4	4	4	4	4	4	4	5	4	4	4
27	0	2	2	8	2	2	1	1	2	5	5	5	4	4	5	5	4	4	4	4	4	4	4	5	4	4	3
28	0	2	1	9	2	1	2	2	1	5	5	5	4	5	5	5	4	4	5	5	5	4	5	5	4	5	5
29	0	2	2	8	2	1	1	1	1	5	4	4	4	4	5	4	4	4	5	4	4	4	4	4	5	3	3
30	0	2	2	5	2	1	1	1	1	4	4	4	4	4	4	4	4	4	4	4	4	4	4	5	5	4	2
31	1	2	2	9	-99	-99	-99	-99	-99	-99	-99	-99	-99	-99	-99	-99	-99	-99	-99	-99	-99	-99	-99	-99	-99	-99	-99
32	0	2	2	8	2	2	1	1	2	4	4	4	4	4	4	4	4	4	4	4	4	4	4	4	4	3	3
33	0	2	1	7	2	1	1	1	1	4	4	4	4	4	5	4	4	4	4	4	4	4	4	5	5	4	4
34	0	2	2	9	2	2	2	1	2	5	5	5	4	5	5	5	5	4	5	5	5	5	4	5	5	4	5
35	0	2	1	6	1	1	1	1	1	4	4	5	3	4	5	5	5	5	5	5	5	5	4	5	5	3	3
36	0	2	2	8	1	1	1	1	1	4	5	5	5	4	5	5	5	5	5	5	5	4	5	5	5	4	4
37	1	2	2	7	-99	-99	-99	-99	-99	-99	-99	-99	-99	-99	-99	-99	-99	-99	-99	-99	-99	-99	-99	-99	-99	-99	-99
38	0	2	2	8	1	1	1	1	1	5	5	5	5	5	5	5	4	4	5	4	5	5	4	4	5	4	4
39	1	2	1	7	-99	-99	-99	-99	-99	-99	-99	-99	-99	-99	-99	-99	-99	-99	-99	-99	-99	-99	-99	-99	-99	-99	-99
40	0	2	1	10	2	2	2	1	2	4	4	4	3	4	4	5	3	3	4	4	4	2	3	5	5	4	3
41	0	2	1	7	1	1	1	1	1	4	4	4	4	4	5	4	4	4	4	4	4	3	4	5	4	3	4
42	0	2	2	7	2	1	1	1	1	4	4	5	4	4	5	4	4	4	4	3	4	4	4	4	4	3	3
43	0	2	2	8	2	2	2	1	1	4	4	4	4	5	5	5	4	4	4	3	4	3	4	4	4	4	3
44	0	2	1	8	2	1	1	2	1	5	4	5	4	4	5	5	4	4	5	5	5	5	4	5	5	5	5
45	0	2	1	8	2	1	1	2	2	4	5	5	4	4	5	5	4	4	5	5	4	4	4	5	5	5	4
46	0	2	1	7	2	1	2	1	1	4	4	4	4	4	5	5	4	4	4	5	5	4	4	5	5	4	4
47	0	2	1	6	2	1	1	1	1	4	4	4	4	4	5	5	5	5	4	4	4	4	4	5	3	4	4
48	0	2	1	7	1	1	1	1	1	4	4	4	3	4	5	4	3	3	4	4	4	4	4	5	5	4	3
49	1	2	1	9	-99	-99	-99	-99	-99	-99	-99	-99	-99	-99	-99	-99	-99	-99	-99	-99	-99	-99	-99	-99	-99	-99	-99
50	0	2	1	7	1	1	1	1	1	4	4	3	4	4	5	4	4	4	4	4	4	4	4	4	4	3	3
51	1	2	1	8	-99	-99	-99	-99	-99	-99	-99	-99	-99	-99	-99	-99	-99	-99	-99	-99	-99	-99	-99	-99	-99	-99	-99

52	0	2	2	8	2	1	1	1	1	4	5	4	4	4	5	4	3	3	4	4	4	4	4	4	3	3	3
53	1	2	1	7	-99	-99	-99	-99	-99	-99	-99	-99	-99	-99	-99	-99	-99	-99	-99	-99	-99	-99	-99	-99	-99	-99	-99
54	0	2	1	8	2	1	1	1	1	5	5	5	4	5	5	5	4	5	5	5	5	4	4	5	5	4	2
55	0	2	1	7	1	1	1	1	1	4	4	5	4	4	5	5	4	4	5	4	4	4	4	5	5	5	5
56	0	2	1	8	1	1	1	1	1	5	3	4	4	4	5	4	4	4	4	3	4	2	4	5	5	3	3
57	0	2	2	10	2	1	1	1	1	4	4	4	3	4	4	4	4	4	4	4	5	4	5	5	5	4	4
58	0	2	2	7	2	2	2	2	1	5	5	5	5	5	5	5	5	5	5	5	4	4	5	5	5	5	5
59	0	2	2	8	2	2	1	1	2	4	5	5	5	5	5	5	5	5	5	5	5	5	4	5	5	5	5
60	1	2	2	8	-99	-99	-99	-99	-99	-99	-99	-99	-99	-99	-99	-99	-99	-99	-99	-99	-99	-99	-99	-99	-99	-99	-99
61	0	2	2	7	2	2	2	2	2	5	4	4	4	4	5	5	4	4	4	4	4	3	3	5	5	5	4
62	0	2	1	5	1	2	2	1	1	5	4	5	4	5	5	4	4	4	4	2	5	4	4	5	4	4	3
63	0	2	2	8	2	2	2	1	2	5	5	5	5	5	5	5	4	4	5	5	4	4	4	5	5	5	5
64	0	2	2	9	1	1	1	1	1	5	4	4	4	4	5	4	4	4	4	4	4	4	4	5	4	4	4
65	0	2	1	10	1	2	2	1	2	4	5	4	4	4	5	5	4	4	5	4	4	4	4	4	5	3	3
66	1	2	1	9	-99	-99	-99	-99	-99	-99	-99	-99	-99	-99	-99	-99	-99	-99	-99	-99	-99	-99	-99	-99	-99	-99	-99
67	0	2	1	7	2	2	2	1	2	5	5	4	3	3	5	4	2	3	3	4	4	3	4	5	5	5	5
68	0	2	1	8	1	1	1	1	1	4	4	4	3	3	4	4	3	3	4	4	4	4	4	4	3	3	3
69	0	2	1	7	1	1	1	1	1	4	4	4	4	4	4	4	4	4	4	4	4	3	3	4	5	4	4
70	1	2	1	7	-99	-99	-99	-99	-99	-99	-99	-99	-99	-99	-99	-99	-99	-99	-99	-99	-99	-99	-99	-99	-99	-99	-99
71	1	2	1	7	-99	-99	-99	-99	-99	-99	-99	-99	-99	-99	-99	-99	-99	-99	-99	-99	-99	-99	-99	-99	-99	-99	-99
72	0	2	1	7	1	1	2	1	1	4	4	5	4	4	5	5	4	3	5	5	4	4	4	5	5	4	3
73	0	2	1	7	1	1	1	1	1	4	4	4	4	3	5	5	4	3	4	4	4	3	4	5	5	5	5
74	1	2	2	7	-99	-99	-99	-99	-99	-99	-99	-99	-99	-99	-99	-99	-99	-99	-99	-99	-99	-99	-99	-99	-99	-99	-99
75	0	2	2	7	2	1	1	1	1	4	4	4	4	4	4	4	2	3	4	4	4	4	3	5	5	4	3
76	0	2	2	9	1	2	2	1	2	5	5	5	4	4	5	5	5	5	5	4	4	4	4	5	5	5	4
77	0	2	1	9	1	1	1	1	1	4	4	5	4	5	5	5	4	4	4	4	4	3	4	5	4	4	3
78	0	2	1	7	2	1	1	2	1	4	4	4	3	4	4	5	3	3	4	4	4	3	4	5	5	3	3
79	0	2	2	7	2	2	1	1	1	4	4	4	4	4	4	4	3	3	4	4	4	4	4	5	5	4	3
80	0	2	1	8	2	1	1	1	1	4	4	4	4	4	4	4	4	4	4	4	4	4	4	5	5	3	4
81	0	2	1	9	2	2	2	2	2	4	4	5	4	4	5	5	4	4	4	4	4	3	4	5	4	4	3
82	0	2	1	8	1	1	1	1	1	4	5	4	3	3	5	4	4	4	5	4	5	4	4	4	5	3	2
