# Supplementary material for: Presentation and evaluation of a modern course in disaster medicine and humanitarian assistance for medical students
Source: BMC Med Educ. 2021 Dec 10;21:610. doi: 10.1186/s12909-021-03043-6 (PMC8661312; doi:10.1186/s12909-021-03043-6)
Supplement: Supplementary file 3 — Additional file 3. [file 12909_2021_3043_MOESM3_ESM.pdf]

Vorschau

Pre-Test

Datum: 14. Apr 2021, 21:14 Maximale Punktezahl: 30

Frage 1 - Naturkatastrophen (1 Punkt)

Die häufigsten Naturkatastrophen im letzten Jahrzehnt waren:

Bitte wählen Sie maximal 1 von 4 Antworten!

- [ ] Überschemmungen
- [ ] Stürme
- [ ] Erdbeben
- [ ] Hitzewellen

Frage 2 - Man-Made Katastrophen (1 Punkt)

Zu Man-Made Katastrophen zählen:

Bitte wählen Sie maximal 1 von 4 Antworten!

- [ ] Dürren
- [ ] Kriege
- [ ] Hitzewellen
- [ ] Schädlingsbefall

Frage 3 - MANV Stufe (1 Punkt)

Was trifft auf MANV Stufe 3 zu?

Bitte wählen Sie maximal 1 von 4 Antworten!

- [ ] Definitionsgemäß maximal 11-25 Verletzte
- [ ] Ist gekennzeichnet durch eine wesentliche Zerstörung der örtlichen Infrastruktur
- [ ] Ist mit Kräften des Regelrettungsdienstes problemlos zu bewältigen
- [ ] Beinhaltet den Einsatz von ehrenamtlichen Rettungskräften neben Kräften des Regelrettungsdienstes

Frage 4 - Aufgaben LNA (1 Punkt)

Zu den Aufgaben des Leitenden Notarztes zählen **nicht:**

Bitte wählen Sie maximal 1 von 4 Antworten!

- ☐ Die direkte medizinische Patientenversorgung
- ☐ Die Beurteilung der Lage aus medizinischer Sicht
- ☐ Die Festlegung von Behandlungs- und Transportprioritäten
- ☐ Weisungsbefugnis gegenüber allen medizinischen Mitarbeiten

Frage 5 - Großere Schadenlagen (1 Punkt)

Bei größeren Schadenslagen ist es wichtig:

Bitte wählen Sie maximal 1 von 4 Antworten!

- ☐ Alle Patienten so schnell wie möglich in das nächste Krankenhaus zu verbringen
- ☐ Transportkapazitäten voll auszunutzen und Leicht- wie Schwerverletze gleichzeitig zu transportieren
- ☐ Eine Verteilung der Patienten in verschiedene geeignete Krankenhäuser zu sichern
- ☐ Eine genaue Erfassung der Patientendaten und Transportziele ist aufgrund des Zeitaufwands nicht notwendig

Frage 6 - Ankunft, Analyse und Lagemeldung (1 Punkt)

Zur Ankunft, Analyse und Lagemeldung gehört **nicht**

Bitte wählen Sie maximal 1 von 5 Antworten!

- ☐ Briefing durch den Gesamteinsatzleiter (Polizei/Feuerwehr, etc.) vor Ort
- ☐ Die Erfassung der Windrichtung
- ☐ Die Erfassung der möglichen Zu- und Abfahrtsrouten
- ☐ Die Beurteilung der Örtlichkeiten zum Einrichten möglicher Behandlungsplätze
- ☐ Die exakte Triage aller Betroffenen

Frage 7 - Verletztes Teammitglied (1 Punkt)

Welche Aussage trifft **nicht** zu? Bei Verletzung eines Teammitglieds:

Bitte wählen Sie maximal 1 von 4 Antworten!

- ☐ Hat es keine Auswirkungen auf das Rettungsteam
- ☐ Können die Betroffenen ggf. nicht mehr richtig versorgt werden
- ☐ Ist der gesamte Einsatz / Mission gefährdet
- ☐ Nimmt ggf. die komplette Organisation Schaden

Frage 8 - Funken (1 Punkt)

Welche Aussage trifft **nicht** zu? Beim Funken ist es wichtig auf folgendes zu achten:

Bitte wählen Sie maximal 1 von 4 Antworten!

- ☐ Laufende Gespräche nicht unterbrechen
- ☐ Komplizierte Worte zu buchstabieren
- ☐ Es wird immer zuerst der Sendende Gesprächsteilnehmer genannt
- ☐ Sollte der Gesprächspartner nicht antworten, wird der Anruf 3x wiederholt und dann blind abgesetzt

Frage 9 - Triage Algorithmus (1 Punkt)

Welche Aussage zum START/mSTART-Algorithmus trifft **nicht** zu?

Bitte wählen Sie maximal 1 von 4 Antworten!

- ☐ Das START Triage-System ist das am häufigsten im zivilen verwendete Triage-System
- ☐ Die Bedeutung ist Simple Triage And Rapid Treatment
- ☐ Die einzige Behandlung, die während der Triage erlaubt ist, ist die Reanimation
- ☐ Falls der Betroffene keine Spontanatmung zeigt, sollten die Atemwege freigemacht und der Kopf rekliniert werden

Frage 10 - Sichtung (1 Punkt)

Welche Aussage zur Sichtung trifft **nicht** zu?

Bitte wählen Sie maximal 1 von 4 Antworten!

- ☐ Sind Kinder in derselben Sichtungskategorie wie Erwachsene, werden diese vor den Erwachsenen behandelt.
- ☐ Kinder sind automatisch in eine dringendere Sichtungskategorie als Erwachsene einzuordnen
- ☐ Im jumpSTART Algorithmus sind 5 initiale Beatmungen bei fehlender Spontanatmung und tastbaren Pulsen notwendig
- ☐ Der neurologische Status kann bei Kindern über das AVPU-Schema bewertet werden

Frage 11 - Dekontamination (1 Punkt)

Dekontamination...

Bitte wählen Sie maximal 1 von 4 Antworten!

- ☐ findet ausschließlich am Schadensort statt
- ☐ ist in den umliegenden Kliniken nicht notwendig
- ☐ sollte auch in Zielkliniken durchgeführt werden können

- [ ] wird zum größten Teil nicht durch die Entkleidung der Betroffenen erreicht

Frage 12 - CBRN (1 Punkt)

Im Fall einer möglichen Kontamination mit CBRN-Stoffen gilt **nicht**

Bitte wählen Sie maximal 1 von 4 Antworten!

- [ ] Die grüne Zone muss mit einem Abstand von mindestens 200m festgelegt werden
- [ ] Die rote Zone stellt die Gefahrenzone, ggf. mit Explosionsgefahr, dar
- [ ] Betroffene (ggf. kontaminierte Patienten) verbleiben bis zur sicheren Dekontamination in der gelben Zone
- [ ] Lebensgefährlich verletzte Patienten werden, unabhängig von einer möglichen Kontamination, sofort nach Ankunft des Rettungsdienstes behandelt.

Frage 13 - Stress (1 Punkt)

Welche Aussage trifft zu?

Bitte wählen Sie maximal 1 von 5 Antworten!

- [ ] In einer Katastrophensituation gibt es nur wenige Stressoren, welche einen psychologischenSupport indizieren könnten.
- [ ] Stressreaktionen gibt es nur auf körperlicher Ebene.
- [ ] Einsatzbewältigung kann erst nach einem Einsatz stattfinden.
- [ ] Während des Einsatzes sind keine Pausen aus Gründen der Einsatzbewältigung möglich.
- [ ] Gespräche zur Einsatz- und Notfallnachsorge sind auf freiwilliger Basis.

Frage 14 - Kapazitätserweiterung (1 Punkt)

Welche Aussage trifft für die Kapazitätserweiterung bei der Klinischen Versorgung im Katastrophenfall **nicht** zu?

Bitte wählen Sie maximal 1 von 4 Antworten!

- [ ] Schaffung zusätzlicher Intensivbehandlungsplätze in Aufwacheinheiten/ Intermediate-Care-Bereichen
- [ ] Freimachen von regulären Intensivbehandlungsbetten durch Verlegung auf Normalstationen oder andere Krankenhäuser
- [ ] Schaffung zusätzlicher OP-Kapazitäten in Notaufnahmen/Schockräumen / Behandlungsräumen
- [ ] Rekrutierung der REA-Teams der Intensivstationen für die Versorgung der Betroffenen

Frage 15 - Interne Lage (1 Punkt)

Was gehört **nicht** zu einer internen Katastrophenlage in einer Klinik?

Bitte wählen Sie maximal 1 von 5 Antworten!

- Bombendrohung
- Ausfall der materiellen Logistik
- Massenkarambolage
- Brand
- Freiwerden von Gasen

Frage 16 - ten priorities (1 Punkt)

Zu den „ten priorities after disasters" zählt **nicht:**

Bitte wählen Sie maximal 1 von 4 Antworten!

- Zelte und Notunterkünfte
- Medizinische Versorgung
- Kontrolle von Epidemien
- Mediale Aufmerksamkeit

Frage 17 - Lage Flüchtlingscamps (1 Punkt)

Kriterium für die Auswahl einer optimalen Fläche für ein Flüchtlingscamp ist **nicht:**

Bitte wählen Sie maximal 1 von 4 Antworten!

- Die Sicherheit in der Umgebung
- Die Lage auf einem Hügel
- Der Zugang zu sauberem Wasser
- Eine gute Erreichbarkeit

Frage 18 - Verantwortung zur Hilfeleistung (1 Punkt)

Die Verantwortung zur Hilfeleistung nach einer nationalen Katastrophe hat an erster Stelle:

Bitte wählen Sie maximal 1 von 4 Antworten!

- Der jeweilige Staat in welchem die Katastrophe stattfindet
- Die Vereinten Nationen
- UN-OCHA
-

Das Internationale Komitee vom Roten Kreuz

Frage 19 - Humanitäre Prinzipien (1 Punkt)

Zu den Humanitären Prinzipien zählt:

Bitte wählen Sie maximal 1 von 5 Antworten!

- ☐ Humanität
- ☐ Neutralität
- ☐ Unparteilichkeit
- ☐ Unabhängigkeit
- ☐ Alles von A-D richtig

Frage 20 - Sphere (1 Punkt)

Was trifft **nicht** zu? Das Sphere Book…

Bitte wählen Sie maximal 1 von 4 Antworten!

- ☐ Folgt der Philosophie, dass jeder Mensch das Recht hat in Würde zu leben
- ☐ Ist verpflichtend für alle Hilfsorganisationen
- ☐ Wurde nach dem Genozid in Ruanda ins Leben gerufen
- ☐ Wird von einem Zusammenschluss der wichtigsten Hilfsorganisationen herausgegeben

Frage 21 - Patient 1 (1 Punkt)

Sichten Sie den folgenden Patienten mit dem START Algorithmus
Rot: Lebensgefährlich verletzt
Gelb: Schwerverletzt
Grün: Leichtverletzt
Schwarz: Verstorben/momentan nicht behandelbar

30 Jahre männlich
Kopfplatzwunde
Ansprechbar, Verwirrt, folgt Aufforderungen nicht
RR 160/90
HF 100
Normale Atmung

- ☐ Rot
- ☐ Gelb
- ☐ Grün
- ☐

Schwarz

Frage 22 - Patient 2 (1 Punkt)

Sichten Sie den folgenden Patienten mit dem START Algorithmus
Rot: Lebensgefährlich verletzt
Gelb: Schwerverletzt
Grün: Leichtverletzt
Schwarz: Verstorben/momentan nicht behandelbar

80 Jahre weiblich

Brustschmerz
Keine äußerlichen Verletzungen
RR 150/100
HF 120
AF 20/min
Zu schwach um Aufzustehen
Orientiert

Bitte wählen Sie maximal 1 von 4 Antworten!

- [ ] Rot
- [ ] Gelb
- [ ] Grün
- [ ] Schwarz

Frage 23 - Patient 3 (1 Punkt)

Sichten Sie den folgenden Patienten mit dem START Algorithmus
Rot: Lebensgefährlich verletzt
Gelb: Schwerverletzt
Grün: Leichtverletzt
Schwarz: Verstorben/momentan nicht behandelbar

2 Jahre männlich
Schreit und weint
Gebrochener rechter Unterarm
AF 40/min
Kapillare Reperfusion (CRP) <2s
Läuft seiner Mutter hinterher

Bitte wählen Sie maximal 1 von 4 Antworten!

- [ ] Rot
- [ ] Gelb
- [ ]

- [ ] Grün
- [ ] Schwarz

Frage 24 - Patient 4 (1 Punkt)

Sichten Sie den folgenden Patienten mit dem START Algorithmus
Rot: Lebensgefährlich verletzt
Gelb: Schwerverletzt
Grün: Leichtverletzt
Schwarz: Verstorben/momentan nicht behandelbar

55 Jahre männlich
Pat. Kommt wild gestikulierend mit blutenden oberflächlichen Schnittwunden an Armen und Beinen auf dich zu gerannt und ruft um Hilfe für eine Frau

Bitte wählen Sie maximal 1 von 4 Antworten!

- [ ] Rot
- [ ] Gelb
- [ ] Grün
- [ ] Schwarz

Frage 25 - Patient 5 (1 Punkt)

Sichten Sie den folgenden Patienten mit dem START Algorithmus
Rot: Lebensgefährlich verletzt
Gelb: Schwerverletzt
Grün: Leichtverletzt
Schwarz: Verstorben/momentan nicht behandelbar

32 Jahre, weiblich
Schwanger, 30.SSW
Ansprechbar, sehr aufgeregt
Starke Schmerzen im Bauch
CPR 5 sec
AF 24min

- [ ] Rot
- [ ] Gelb
- [ ] Grün
- [ ] Schwarz

Frage 26 - Patient 6 (1 Punkt)

Sichten Sie die folgende Patientin mit dem START Algorithmus
Rot: Lebensgefährlich verletzt
Gelb: Schwerverletzt
Grün: Leichtverletzt
Schwarz: Verstorben/momentan nicht behandelbar

50 Jahre, weiblich
blass, liegend
Keine Spontanatmung
Offene Thoraxverletzung
--> Nach Reklination Spontanatmung
--> AF 32

Bitte wählen Sie maximal 1 von 4 Antworten!

- ☐ Rot
- ☐ Gelb
- ☐ Grün
- ☐ Schwarz

Frage 27 - Patient 7 (1 Punkt)

Sichten Sie die folgende Patientin mit dem START Algorithmus
Rot: Lebensgefährlich verletzt
Gelb: Schwerverletzt
Grün: Leichtverletzt
Schwarz: Verstorben/momentan nicht behandelbar

20 Jahre männlich
Metallsplitter im Auge, sonst keine Verletzungen
CPR < 2sec
Spontanatmend und orientiert

Bitte wählen Sie maximal 1 von 4 Antworten!

- ☐ Rot
- ☐ Gelb
- ☐ Grün
- ☐ Schwarz

Frage 28 - Patient 8 (1 Punkt)

Sichten Sie den folgenden Patienten mit dem START Algorithmus
Rot: Lebensgefährlich verletzt
Gelb: Schwerverletzt
Grün: Leichtverletzt
Schwarz: Verstorben/momentan nicht behandelbar

45 Jahre männlich
Offen gebrochener Unterschenkel
Wach, orientiert
Spontanatmend
CPR < 2sec

Bitte wählen Sie maximal 1 von 4 Antworten!

- [ ] Rot
- [ ] Gelb
- [ ] Grün
- [ ] Schwarz

Frage 29 - Patient 9 (1 Punkt)

Sichten Sie den folgenden Patienten mit dem START Algorithmus
Rot: Lebensgefährlich verletzt
Gelb: Schwerverletzt
Grün: Leichtverletzt
Schwarz: Verstorben/momentan nicht behandelbar

35 Jahre männlich
Thoraxdurchspießung
Keine Spontanatmung
Nach Reklination keine Spontanatmung

Bitte wählen Sie maximal 1 von 4 Antworten!

- [ ] Rot
- [ ] Gelb
- [ ] Grün
- [ ] Schwarz

Frage 30 - Patient 10 (1 Punkt)

Sichten Sie den folgenden Patienten mit dem START Algorithmus
Rot: Lebensgefährlich verletzt
Gelb: Schwerverletzt
Grün: Leichtverletzt
Schwarz: Verstorben/momentan nicht behandelbar

15 Jahre weiblich
Hämatom linker Oberbauch
AF 50
Steht gekrümmt an einer Säule
Wach und orientiert
CPR 4 sec

Bitte wählen Sie maximal 1 von 4 Antworten!

- [ ] Rot
- [ ] Gelb
- [ ] Grün
- [ ] Schwarz

Vorschau

Post-Test

Datum: 14. Apr 2021, 21:16 Maximale Punktezahl: 30

Frage 1 - Katastrophenkriterien (1 Punkt)

Welches Kriterium zählt nicht, um von den Vereinten Nationen als Katastrophe anerkannt zu werden

Bitte wählen Sie maximal 1 von 5 Antworten!

- ☐ 10 Menschen oder mehr sind bei einem Ereignis verstorben
- ☐ 100 Menschen oder mehr sind von einem Ereignis betroffen
- ☐ Erklärung des Katastrophenfalls der Regierung des Landes
- ☐ Das internationale Hilfsersuchen einer Regierung
- ☐ Das Hilfsersuchen eines einzelnen Betroffenen

Frage 2 - Todesfälle Naturkatastrophen (1 Punkt)

Die meisten Todesfälle bei Naturkatastrophen im letzten Jahrzehnt gab es bei

Bitte wählen Sie maximal 1 von 4 Antworten!

- ☐ Erdbeben
- ☐ Stürmen
- ☐ Hitzewellen
- ☐ Dürren

Frage 3 - Prognose (1 Punkt)

In der Katastrophenmedizin zählt folgende Prognose

Bitte wählen Sie maximal 1 von 2 Antworten!

- ☐ Individualprognose
- ☐ Gesamtprognose

Frage 4 - Einsatzleitung (1 Punkt)

Wer hat bei größeren Schadenslagen mit Gefahrgut unterhalb der Katastrophenschwelle die Gesamteinsatzleitung vor Ort

Bitte wählen Sie maximal 1 von 5 Antworten!

- ☐ Derjenige, der am lautesten schreit
- ☐ Der Rettungsdienstleiter
- ☐ Der Landrat/Oberbürgermeister
- ☐ Der örtliche Einsatzleiter der Feuerwehr
- ☐ Leitender Notarzt und organisatorischer Leiter Rettungsdienst

Frage 5 - OrgL (1 Punkt)

Welche Aussage trifft nicht zu: Der „OrgL“

Bitte wählen Sie maximal 1 von 4 Antworten!

- ☐ ist der Organisatorische Leiter Rettungsdienst
- ☐ ist vor allem für die technisch – organisatorische Beurteilung der medizinischen Lage verantwortlich
- ☐ ist in Baden-Württemberg mit einer pinken Weste gekennzeichnet
- ☐ ist für die Kommunikation mit der Leitstelle zuständig

Frage 6 - METHANE Report (1 Punkt)

Der sogenannte METHANE Report gibt **keinen** Aufschluss über

Bitte wählen Sie maximal 1 von 4 Antworten!

- ☐ die erste Lageeinschätzung auf Sicht
- ☐ die ausführliche 2.Lagemeldung
- ☐ Zu- und Abfahrtswege
- ☐ die ungefähre Anzahl der Betroffenen

Frage 7 - PHTLS (1 Punkt)

Beim PHTLS (Pre-Hospital-Trauma-Life-Support) ist die folgende Zuordnung falsch

Bitte wählen Sie maximal 1 von 5 Antworten!

- ☐ A Airway
- ☐ B Breathing
- ☐ C Circulation
- ☐ D Disaster
- ☐ E Environment

Frage 8 - Katastropheneinsatz (1 Punkt)

Bei einem Katastropheneinsatz ist es das Wichtigste

Bitte wählen Sie maximal 1 von 4 Antworten!

- ☐ dem einzelnen Patienten zu helfen
- ☐ die Vorgaben der Hilfsorganisation zu erfüllen
- ☐ der Eigenschutz des Einsatzteams
- ☐ die mediale Wirkung auf das Ereignis zu lenken

Frage 9 - Kommunikation (1 Punkt)

Welche Aussage zur Kommunikation am Einsatzort trifft zu?

Bitte wählen Sie maximal 1 von 4 Antworten!

- ☐ Mobiltelefone sind ausfallssicher und stehen auch in Katastrophenfällen jederzeit zur Verfügung
- ☐ Satellitentelefone sind flächendeckend verfügbar und überall auf der Welt ausfallssicher
- ☐ Bei größeren Schadenslagen werden regelhaft Feldtelefone (kabelgebunden) etabliert
- ☐ Am Einsatzort ist die Kommunikation über BOS-Funkgeräte (2m/4m/TETRA) üblich

Frage 10 - START-Triage (1 Punkt)

Zu den Behandlungen, die während der START-Triage erlaubt sind, zählt **nicht**:

Bitte wählen Sie maximal 1 von 4 Antworten!

- ☐ Tracheotomie
- ☐ Stopp spritzender arterieller Blutungen
- ☐ Entlastung eines Spannungspneumothorax
- ☐ Öffnung der Atemwege

Frage 11 - Blaue Sichtungskategorie (1 Punkt)

Die Blaue Sichtungskategorie

Bitte wählen Sie maximal 1 von 4 Antworten!

- ☐ Wurde in den USA erfunden
- ☐ Beschreibt Patienten mit sicheren Todeszeichen
- ☐ Beschreibt Patienten die noch am Leben sind, denen aber mit den vorhandenen Ressourcen nach Triage Algorithmus aktuell nicht geholfen werden kann
- ☐ Bedeutet eine sofortige Behandlung

Frage 12 - Humanitäre Charta (1 Punkt)

Die Humanitäre Charta beinhaltet:

Bitte wählen Sie maximal 1 von 4 Antworten!

- ☐ Das Recht auf ein Leben in Würde
- ☐ Das Recht auf Humanitäre Hilfe
- ☐ Alle sind richtig
- ☐ Das Recht auf Schutz und Sicherheit

Frage 13 - Schlüsselaspekte der Sichtung (1 Punkt)

Welche Aussage zu den Schlüsselaspekten der Sichtung trifft nicht zu?

Bitte wählen Sie maximal 1 von 4 Antworten!

- ☐ Triage soll die Gruppe derer identifizieren welche sofortige Hilfe benötigen und ist eine Determinante für das Überleben dieser lebensgefährlich Verletzten
- ☐ Triage soll die effektive Nutzung der vorhandenen Ressourcen sicherstellen.
- ☐ Der Zustand der Betroffenen kann sich im zeitlichen Verlauf verändern und möglicherweise schlagartig verschlechtern.
- ☐ Es gibt ein gültiges Sichtungssystem welches weltweit ausschließlich eingesetzt wird.

Frage 14 - Dekontaminatinon (1 Punkt)

Dekontamination...

Bitte wählen Sie maximal 1 von 4 Antworten!

- ☐ findet ausschließlich am Schadensort statt
- ☐ ist in den umliegenden Kliniken nicht notwendig
- ☐ sollte auch in Zielkliniken durchgeführt werden können
- ☐ wird zum größten Teil nicht durch die Entkleidung der Betroffenen erreicht

Frage 15 - Psychologischer Support (1 Punkt)

Was gehört **nicht** zum psychologischen Support bei Katastrophen?

Bitte wählen Sie maximal 1 von 5 Antworten!

- ☐ Einsatzbewältigung
- ☐ Tiefenpsychologische Deutung nach Großschadensfällen
- ☐ Primäre Prävention
- ☐

Einsatznachsorge

- [ ] Notfallnachsorge

Frage 16 - Surge Capacity (1 Punkt)

Was gehört **nicht** zu den 4S der Surge Capacity?

Bitte wählen Sie maximal 1 von 4 Antworten!

- [ ] Symbols
- [ ] Systems
- [ ] Staff
- [ ] Structure

Frage 17 - Ten priorities (1 Punkt)

Zu den „ten priorities after disasters“ zählt **nicht:**

Bitte wählen Sie maximal 1 von 4 Antworten!

- [ ] Mediale Aufmerksamkeit
- [ ] Masern Impfungen
- [ ] Wasser und Sanitär
- [ ] Nahrungsmittelsicherheit

Frage 18 - Epidemien (1 Punkt)

Zu den häufigen Epidemien nach Katastrophen zählen **nicht**

Bitte wählen Sie maximal 1 von 4 Antworten!

- [ ] Hepatitis B
- [ ] Malaria
- [ ] Cholera
- [ ] Masern

Frage 19 - UN Organisationen (1 Punkt)

Die UN-Organisation, die auf die Koordinierung der Humanitären Hilfsmaßnahmen nach einer internationalen Katastrophe spezialisiert ist, heißt:

Bitte wählen Sie maximal 1 von 4 Antworten!

- [ ] ICRC
- [ ] UN-HCR

- UN-OCHA
- Unicef

Frage 20 - Wasser (1 Punkt)

Nach dem WASH Standard benötigt jeder Mensch täglich mindestens:

Bitte wählen Sie maximal 1 von 4 Antworten!

- 40 Liter Wasser
- 15 Liter Wasser
- 5 Liter Wasser
- 30 Liter Wasser

Frage 21 - Triage 1 (1 Punkt)

Sichten Sie die folgenden Patienten nach START Triage
Rot: Lebensgefährlich verletzt
Gelb: Schwerverletzt
Grün: Leichtverletzt
Schwarz: Verstorben

30 Jahre weiblich

Kopfverletzung blutend, blasses Hautkolorit, nicht gehfähig

RR 160/90
HF 100
Normale Atmung

Ansprechbar, Verwirrt, folgt Aufforderungen nicht

Bitte wählen Sie maximal 1 von 4 Antworten!

- Grün
- Gelb
- Rot
- Schwarz

Frage 22 - Triage 2 (1 Punkt)

Sichten Sie die folgenden Patienten nach START Triage
Rot: Lebensgefährlich verletzt
Gelb: Schwerverletzt
Grün: Leichtverletzt
Schwarz: Verstorben

70 Jahre weiblich
Brustschmerz
Keine äußerlichen Verletzungen
CPR <2sec
HF 120
AF 20/min
Zu schwach um Aufzustehen, nicht gehfähig
Orientiert

Bitte wählen Sie maximal 1 von 4 Antworten!

- [ ] Grün
- [ ] Gelb
- [ ] Rot
- [ ] Schwarz

Frage 23 - Triage 3 (1 Punkt)

Sichten Sie die folgenden Patienten nach START Triage
Rot: Lebensgefährlich verletzt
Gelb: Schwerverletzt
Grün: Leichtverletzt
Schwarz: Verstorben

4 Jahre weiblich
Schreit und weint
Verletzung am rechten Auge, kann nichts sehen, weint, redet mit seiner Mutter
AF 44/min
Kapillare Reperfusion (CRP) <2s
gehfähig

Bitte wählen Sie maximal 1 von 4 Antworten!

- [ ] Grün
- [ ] Gelb
- [ ] Rot
- [ ] Schwarz

Frage 24 - Triage 4 (1 Punkt)

Sichten Sie die folgenden Patienten nach START Triage
Rot: Lebensgefährlich verletzt
Gelb: Schwerverletzt
Grün: Leichtverletzt
Schwarz: Verstorben

Pat. Kommt wild gestikulierend mit blutenden oberflächlichen Schnittwunden an Armen und Beinen auf dich zu gerannt und ruft um Hilfe für eine Frau

Bitte wählen Sie maximal 1 von 4 Antworten!

- [ ] Grün
- [ ] Gelb
- [ ] Rot
- [ ] Schwarz

Frage 25 - Triage 5 (1 Punkt)

Sichten Sie die folgenden Patienten nach START Triage
Rot: Lebensgefährlich verletzt
Gelb: Schwerverletzt
Grün: Leichtverletzt
Schwarz: Verstorben

32 Jahre, weiblich

Schwanger, 30.SSW
Ansprechbar, sehr aufgeregt
Starke Schmerzen im Bauch
CPR 5 sec
AF 24/min

Bitte wählen Sie maximal 1 von 4 Antworten!

- [ ] Grün
- [ ] Gelb
- [ ] Rot
- [ ] Schwarz

Frage 26 - Triage 6 (1 Punkt)

Sichten Sie die folgenden Patienten nach START Triage
Rot: Lebensgefährlich verletzt
Gelb: Schwerverletzt
Grün: Leichtverletzt
Schwarz: Verstorben

36 Jahre, weiblich

blass, liegend
Keine Spontanatmung
Offene Thoraxverletzung

--> Nach Reklination Spontanatmung
--> AF 32/min

Bitte wählen Sie maximal 1 von 4 Antworten!

- [ ] Grün
- [ ] Gelb
- [ ] Rot
- [ ] Schwarz

Frage 27 - Triage 7 (1 Punkt)

Sichten Sie die folgenden Patienten nach START Triage
Rot: Lebensgefährlich verletzt
Gelb: Schwerverletzt
Grün: Leichtverletzt
Schwarz: Verstorben

20 Jahre weiblich
26.SSW, gespanntes Abdomen, starke Schmerzen, nicht gehfähig

AF 28/min
CPR 4sec
Spontanatmend und orientiert

Bitte wählen Sie maximal 1 von 4 Antworten!

- [ ] Grün
- [ ] Gelb
- [ ] Rot
- [ ] Schwarz

Frage 28 - Triage 8 (1 Punkt)

Sichten Sie die folgenden Patienten nach START Triage
Rot: Lebensgefährlich verletzt
Gelb: Schwerverletzt
Grün: Leichtverletzt
Schwarz: Verstorben

31 Jahre männlich
Offen gebrochener Unterschenkel, nicht gehfähig
Wach, orientiert
Spontanatmend

AF 27/min
CPR < 2sec

Bitte wählen Sie maximal 1 von 4 Antworten!

- Grün
- Gelb
- Rot
- Schwarz

Frage 29 - Triage 9 (1 Punkt)

Sichten Sie die folgenden Patienten nach START Triage
Rot: Lebensgefährlich verletzt
Gelb: Schwerverletzt
Grün: Leichtverletzt
Schwarz: Verstorben

35 Jahre weiblich
Amputation rechter Unterarm

Wach, ansprechbar und orientiert, zu schwach aufzustehen

Arterielle Blutung steht nach Tourniquet-Anlage

AF 36/min

CPR 4sec

Bitte wählen Sie maximal 1 von 4 Antworten!

- Grün
- Rot
- Gelb
- Schwarz

Frage 30 - Triage 10 (1 Punkt)

Sichten Sie die folgenden Patienten nach START Triage
Rot: Lebensgefährlich verletzt
Gelb: Schwerverletzt
Grün: Leichtverletzt
Schwarz: Verstorben

38 Jahre männlich
Liegt auf dem Bauch, keine Sensomotorik in der Unteren Extremität
AF 16
Wach und orientiert
CPR 2 sec

Bitte wählen Sie maximal 1 von 4 Antworten!

- [ ] Grün
- [ ] Gelb
- [ ] Rot
- [ ] Schwarz
