# Supplementary material for: Presentation and evaluation of a modern course in disaster medicine and humanitarian assistance for medical students
Source: BMC Med Educ. 2021 Dec 10;21:610. doi: 10.1186/s12909-021-03043-6 (PMC8661312; doi:10.1186/s12909-021-03043-6)
Supplement: Supplementary file 4 — Additional file 4. [file 12909_2021_3043_MOESM4_ESM.pdf]

Supplemental Material

Raw Data Objective Measurement of Knowledge - Results of the Compulsory Multiple Choice Test of Knowledge

Testscore_pre	Testscore_post
13	30
11	30
18	30
22	30
20	29
21	30
24	30
26	30
19	26
25	30
19	30
22	29
23	30
19	29
21	30
21	30
21	30
22	30
25	30
26	28
25	29
24	29
17	27
25	30
25	28
22	29
23	29
22	25
27	29
29	28
26	29
27	27
21	30
25	29
20	28
23	29
15	30
27	29
22	29
27	29
19	27
22	29
19	29
20	28
19	28
20	28
22	29
17	29
19	28
27	29
20	29
27	30
21	27
26	30
26	28
22	28
23	27
23	26
23	29
21	30
20	30
23	30
25	28
28	29
17	29
21	29
22	30
25	30
25	28
21	29
25	29
28	27
22	28
23	30
22	28
22	29
21	25
22	29
19	29
20	29
19	30
21	28
20	26
22	29
27	25
25	28
23	28
19	28
20	27
23	29
9	22
22	28
20	28
19	29
23	27
23	30
20	25
21	28
22	28
20	29
24	28
23	28
