# Supplementary material for: Presentation and evaluation of a modern course in disaster medicine and humanitarian assistance for medical students
Source: BMC Med Educ. 2021 Dec 10;21:610. doi: 10.1186/s12909-021-03043-6 (PMC8661312; doi:10.1186/s12909-021-03043-6)
Supplement: Supplementary file 5 — Additional file 5. [file 12909_2021_3043_MOESM5_ESM.pdf]

# Umfrage Katastrophenmedizin und Humanitäre Hilfe

## Ergebnisse der Umfrage

### Wintersemester 17/18

| | | |
|---|---|---|
| Teilnehmer: | 20 | |
| Ausgefüllt: | 18 | (90%) |

Diese Umfrage wurde mit dem Ostrakon Online Evaluation System indicate2 durchgeführt. Bei Fragen die verwendete Technologie betreffend kontaktieren Sie software@ostrakon.org.

# 1 Zur Veranstaltung

## 1.1 Die Lernziele wurden eindeutig definiert.

Antworten: 18 von 20 - Rücklauf: 90.0% - Durchschnitt: 1.17 - Varianz: 0.14 - Standardabweichung: 0.37

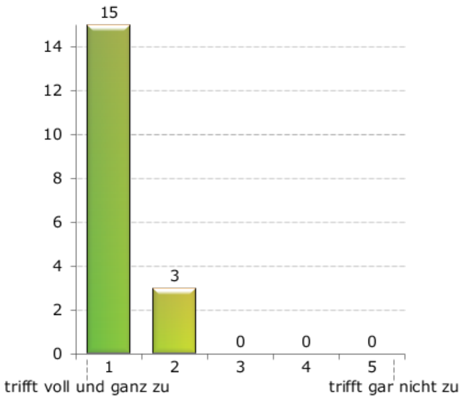


## 1.2 Die Veranstaltung bietet einen guten Überblick über das Themengebiet.

Antworten: 18 von 20 - Rücklauf: 90.0% - Durchschnitt: 1.06 - Varianz: 0.05 - Standardabweichung: 0.23

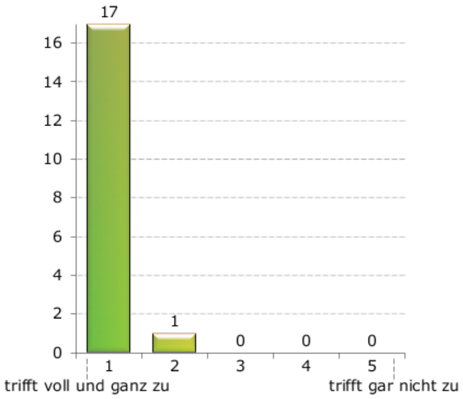


## 1.3 Die Veranstaltung schließt sinnvoll an Lerninhalte anderer Veranstaltungen an.

Antworten: 16 von 20 - Rücklauf: 80.0% - Durchschnitt: 1.25 - Varianz: 0.19 - Standardabweichung: 0.43

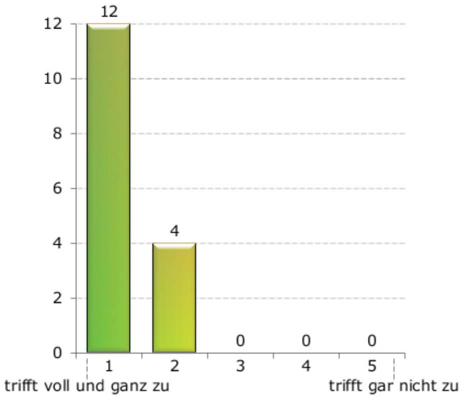

1.4 Die Veranstaltung regt zur Auseinandersetzung mit den behandelten Themen an.

Antworten: 18 von 20 - Rücklauf: 90.0% - Durchschnitt: 1.06 - Varianz: 0.05 - Standardabweichung: 0.23

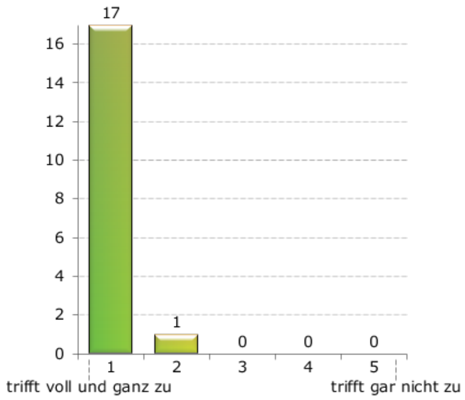


1.5 Die Lehrveranstaltung ist eine gute Mischung aus Wissensvermittlung und Diskussion.

Antworten: 18 von 20 - Rücklauf: 90.0% - Durchschnitt: 1.06 - Varianz: 0.05 - Standardabweichung: 0.23

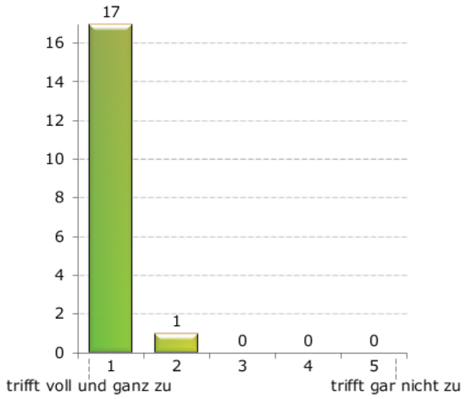


1.6 Mein persönlicher Lernerfolg ist...

Antworten: 18 von 20 - Rücklauf: 90.0% - Durchschnitt: 1.06 - Varianz: 0.05 - Standardabweichung: 0.23

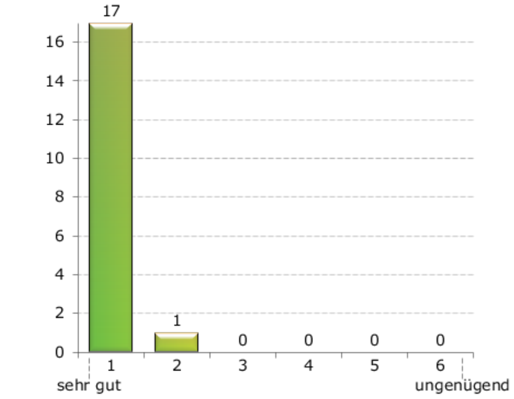

## 1.7 Ich gebe der Veranstaltung die Gesamtbewertung

Antworten: 18 von 20 - Rücklauf: 90.0% - Durchschnitt: 1.00 - Varianz: 0.00 - Standardabweichung: 0.00

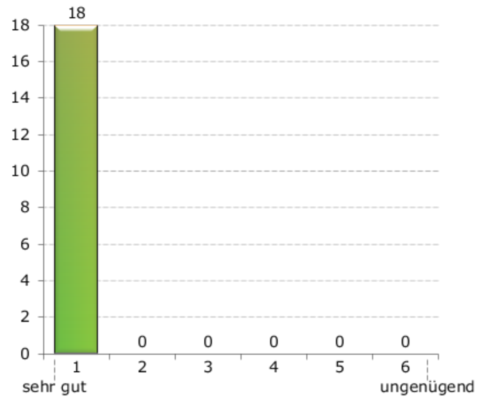


## 1.8 Die Veranstaltung hat pünktlich angefangen.

Antworten: 18 von 20 null 90.0%

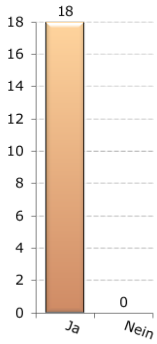


## 1.9 Die Veranstaltung wurde frühzeitig beendet.

Antworten: 17 von 20 null 85.0%

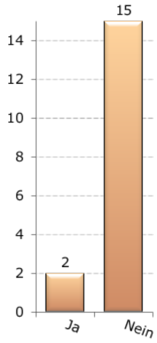


## 1.10 Freie Kommentare :

(sind für die Dozierenden, Studienbeauftragen, Lehrstuhlinhaber und das Studiendekanat einsehbar) Was fanden Sie besonders hilfreich für Ihr Lernergebnis? Was war aus Ihrer Sicht eher hinderlich? Welche konkreten Verbesserungsvorschläge haben Sie?
Antworten: 8 von 20 - Rücklauf: 40.0%

1. Tolles Wahlfach. Die Großübung hat nochmal gut die zuvor gelernten Lerninhalte gefestigt.

2. Beste Veranstaltung des bisherigen Studiums! Vielen Dank dafür! Vielen dank für den großen Einsatz der Dozenten für das Wahlfach, beeindruckend!

3. Das war ein klasse Wahlfach! Besonders gefallen hat mir das breite Spektrum der behandelten Themen und der gute Mix aus aufeinander abgestimmter Theorie und Praxis/Übungen. Der Höhepunkt und die beste Erfahrung war natürlich die Real-Übung, bei der alles zusammengeführt wurde!

4. Tolles Wahlfach ! Wird zu 100% weiterempfohlen.

5. Sehr gute Veranstaltung. Lehrreich, aber mit der nötigen Portion Ernst für die Thematik. Innovative und abwechslungsreiche Lehrmethoden mit gelungener Abschlussübung. Der bisher beste Kurs im Medizinstudium. Weiter so!

6. Das war eine herrausragend gute Veranstaltung! Noch nie habe ich erlebt, dass eine Veranstaltung mit so viel Begeisterung und Herzblut vorbereitet und dann auch durchgeführt wurde. Man hat wirklich die Leidenschaft der Dozenten für diesen Themenbereich miterleben können.
   Die Simulationen mit verschiedenen Computerprogrammen waren super, um in kurzer Zeit die Start-Triage intensiv zu üben. Auch die Übungen zur Thoraxtrainage und zur Koniotomie waren mit den Schweinepräparaten besser als an den üblichen Plastikmodellen.
   Am besten hat mir gefallen, dass man mal aus der "Mediziner-Blase" herausgekommen ist, die anderen Akteure im Katastrophenschutz kennengelernt und mit ihnen zusammengearbeitet hat. Der Besuch bei der Feuerwehr, sowie die Übung mit DRK und THW waren haben dazu eine gute Möglichkeit. Man konnte endlich mal realitätsnah sehen und auch ausprobieren wie im Realfall so eine Zusammenarbeit funktionieren würde. Sonst ist man immer nur unter Medizinern und übt nie mit nicht-Medizinern zusammen. Ich finde, dass so eine Zusammenarbeit auch vielen anderen Lehrveranstaltungen gut tun würde!
   Alles in allem war das ein sehr gelungenens Wahlfach und das mit Abstand beste, das ich bisher besucht habe!
   Ich habe nichts negatives dazu zu äußern. Nur Lob und nochmals ein großes Dankeschön!

7. Super Wahlfach mit perfekter Mischung aus praktischem Bezug und Übung sowie Theorie! Einblicke in das Vorgehen beim Katastrophenfall und die Situation in der humanitären Hilfe waren sehr anschaulich. Es hat wirklich sehr viel Spaß gemacht an diesem aufwändig gestalteten Wahlfach teilzunehmen! Dankeschön für das Wahlfach und all die Mühe, Energie und Ideen, die dahinter stehen!

8. Alles Super

# Umfrage Katastrophenmedizin und Humanitäre Hilfe(1)

## Ergebnisse der Umfrage

### Sommersemester 2018

| | | |
|---|---|---|
| Teilnehmer: | 21 | |
| Ausgefüllt: | 17 | (81%) |

Diese Umfrage wurde mit dem Ostrakon Online Evaluation System indicate2 durchgeführt. Bei Fragen die verwendete Technologie betreffend kontaktieren Sie software@ostrakon.org.

# 1 Zur Veranstaltung

## 1.1 Die Lernziele wurden eindeutig definiert.

Antworten: 16 von 21 - Rücklauf: 76.2% - Durchschnitt: 1.06 - Varianz: 0.06 - Standardabweichung: 0.24

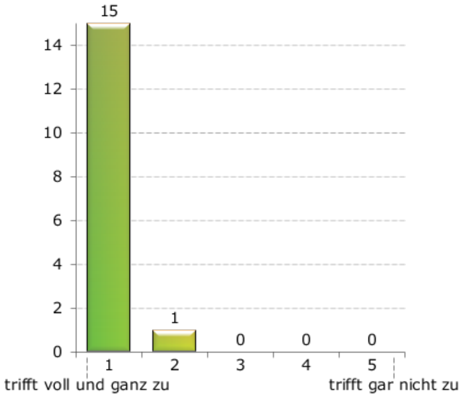


## 1.2 Die Veranstaltung bietet einen guten Überblick über das Themengebiet.

Antworten: 16 von 21 - Rücklauf: 76.2% - Durchschnitt: 1.00 - Varianz: 0.00 - Standardabweichung: 0.00

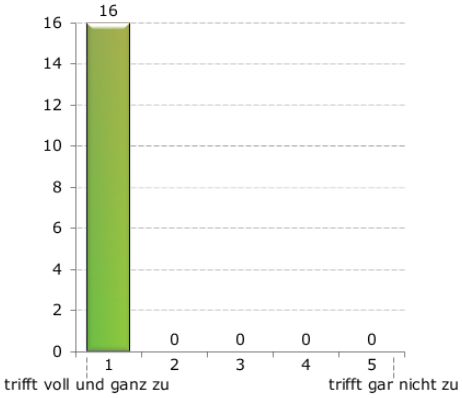


## 1.3 Die Veranstaltung schließt sinnvoll an Lerninhalte anderer Veranstaltungen an.

Antworten: 14 von 21 - Rücklauf: 66.7% - Durchschnitt: 1.64 - Varianz: 1.23 - Standardabweichung: 1.11

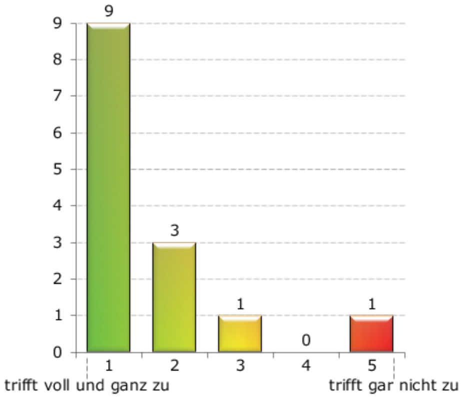

## 1.4 Die Veranstaltung regt zur Auseinandersetzung mit den behandelten Themen an.

Antworten: 16 von 21 - Rücklauf: 76.2% - Durchschnitt: 1.00 - Varianz: 0.00 - Standardabweichung: 0.00

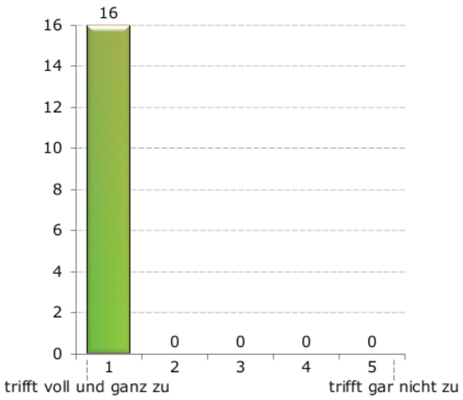


## 1.5 Die Lehrveranstaltung ist eine gute Mischung aus Wissensvermittlung und Diskussion.

Antworten: 16 von 21 - Rücklauf: 76.2% - Durchschnitt: 1.12 - Varianz: 0.11 - Standardabweichung: 0.33

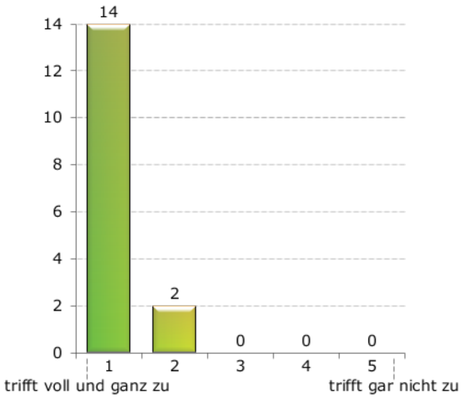


## 1.6 Mein persönlicher Lernerfolg ist...

Antworten: 16 von 21 - Rücklauf: 76.2% - Durchschnitt: 1.00 - Varianz: 0.00 - Standardabweichung: 0.00

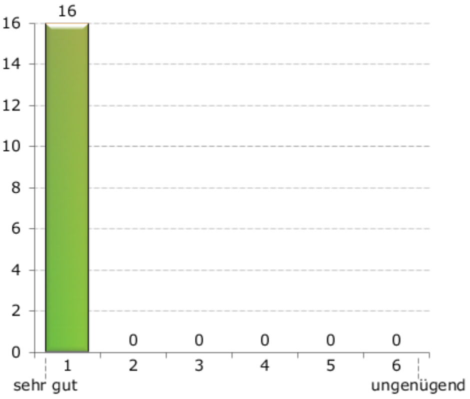

## 1.7 Ich gebe der Veranstaltung die Gesamtbewertung

Antworten: 16 von 21 - Rücklauf: 76.2% - Durchschnitt: 1.00 - Varianz: 0.00 - Standardabweichung: 0.00

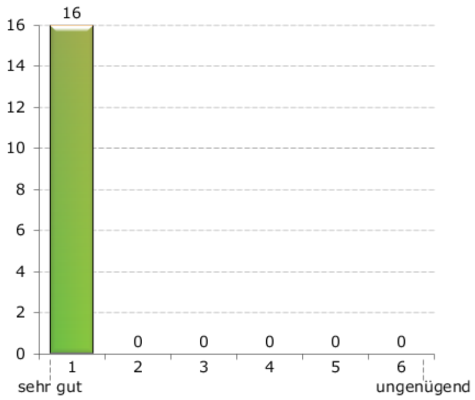


## 1.8 Die Veranstaltung hat pünktlich angefangen.

Antworten: 16 von 21 null 76.2%

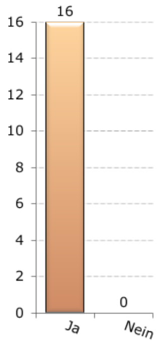


## 1.9 Die Veranstaltung wurde frühzeitig beendet.

Antworten: 15 von 21 null 71.4%

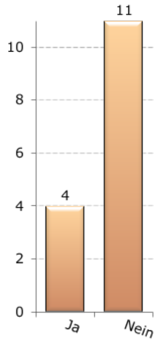


## 1.10 Freie Kommentare :

(sind für die Dozierenden, Studienbeauftragen, Lehrstuhlinhaber und das Studiendekanat einsehbar) Was fanden Sie besonders hilfreich für Ihr Lernergebnis? Was war aus Ihrer Sicht eher hinderlich? Welche konkreten Verbesserungsvorschläge haben Sie?
Antworten: 3 von 21 - Rücklauf: 14.3%

1. Wirklich die beste Veranstaltung, die ich bisher besucht habe! Also kein Vergleich zu den ganzen iklics usw. Man lernt wirklich Dinge, die egal welche Fachrichtung man einschlägt brauchen kann!

2. super Wahlfach, man merkt wie viel Herzblut reingesteckt wurde, hat mich neeindruckt wie gut alles organisiert war obwohl das Ganze erst zum zweiten Mal angeboten wurde, da können sich viele andere Veranstaltungen gerne eine Scheibe abschneiden

3. Super informativer und wichtiger Kurs, der die Themen Katastrophenmedizin und humanitäre Hilfe sachlich, verständlich und anschaulich wiedergibt. Man hat anschließend begriffen, wie sehr uns alle das Fachgebiet betrifft und viele Vorurteile widerlegt. Klasse Team, das mit viel Fleiß ein abwechslungreiches Wahlfach bietet. Die Kontrolle der Abschlussklausur zeigte den eigenen Lernerfolg. Das Themengebiet sollte unbedingt in den Lehrplan mit aufgenommen werden!

# Umfrage Katastrophenmedizin und Humanitäre Hilfe(1)(2)

## Ergebnisse der Umfrage

### Wintersemester 18/19

| | | |
|---|---|---|
| Teilnehmer: | 22 | |
| Ausgefüllt: | 17 | (77%) |

Diese Umfrage wurde mit dem Ostrakon Online Evaluation System indicate2 durchgeführt. Bei Fragen die verwendete Technologie betreffend kontaktieren Sie software@ostrakon.org.

# 1 Zur Veranstaltung

## 1.1 Die Lernziele wurden eindeutig definiert.

Antworten: 17 von 22 - Rücklauf: 77.3% - Durchschnitt: 1.00 - Varianz: 0.00 - Standardabweichung: 0.00

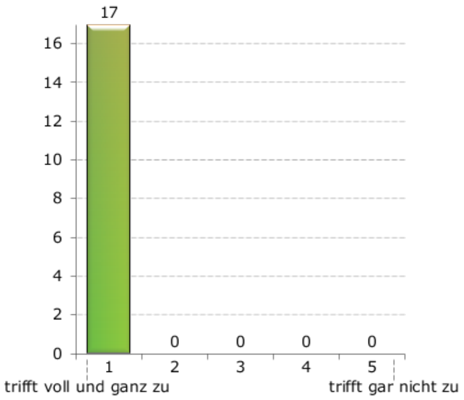


## 1.2 Die Veranstaltung bietet einen guten Überblick über das Themengebiet.

Antworten: 17 von 22 - Rücklauf: 77.3% - Durchschnitt: 1.00 - Varianz: 0.00 - Standardabweichung: 0.00

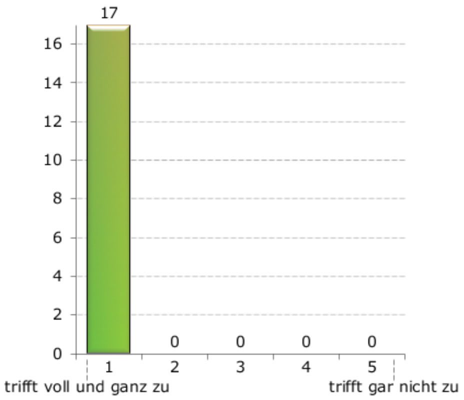


## 1.3 Die Veranstaltung schließt sinnvoll an Lerninhalte anderer Veranstaltungen an.

Antworten: 16 von 22 - Rücklauf: 72.7% - Durchschnitt: 1.19 - Varianz: 0.15 - Standardabweichung: 0.39

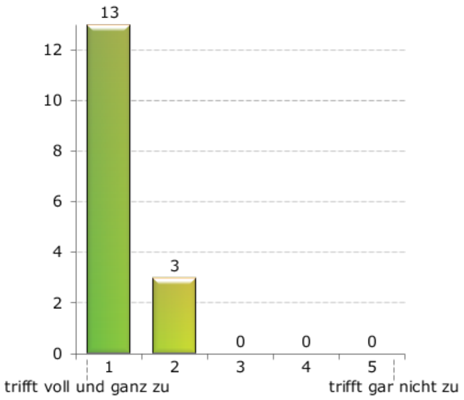

1.4 Die Veranstaltung regt zur Auseinandersetzung mit den behandelten Themen an.

Antworten: 17 von 22 - Rücklauf: 77.3% - Durchschnitt: 1.00 - Varianz: 0.00 - Standardabweichung: 0.00

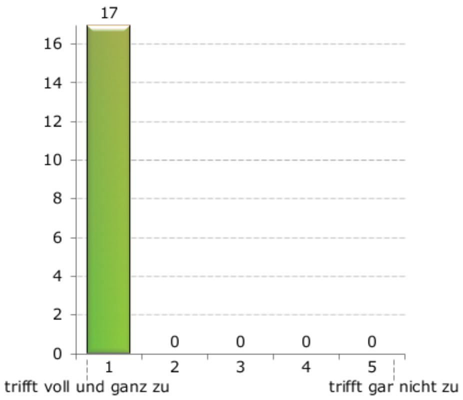


1.5 Die Lehrveranstaltung ist eine gute Mischung aus Wissensvermittlung und Diskussion.

Antworten: 17 von 22 - Rücklauf: 77.3% - Durchschnitt: 1.06 - Varianz: 0.06 - Standardabweichung: 0.24

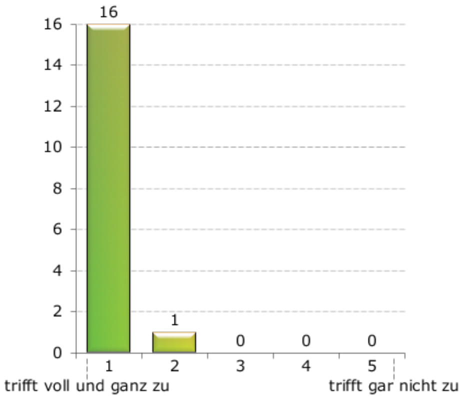


1.6 Mein persönlicher Lernerfolg ist...

Antworten: 17 von 22 - Rücklauf: 77.3% - Durchschnitt: 1.06 - Varianz: 0.06 - Standardabweichung: 0.24

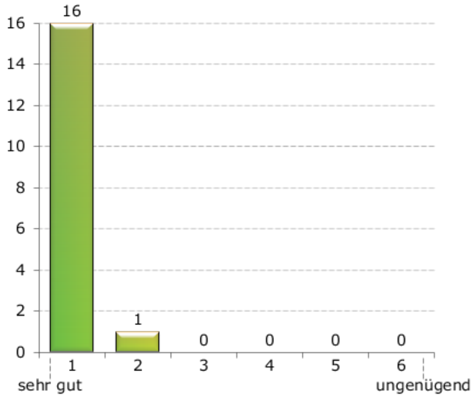

## 1.7 Ich gebe der Veranstaltung die Gesamtbewertung

Antworten: 17 von 22 - Rücklauf: 77.3% - Durchschnitt: 1.00 - Varianz: 0.00 - Standardabweichung: 0.00

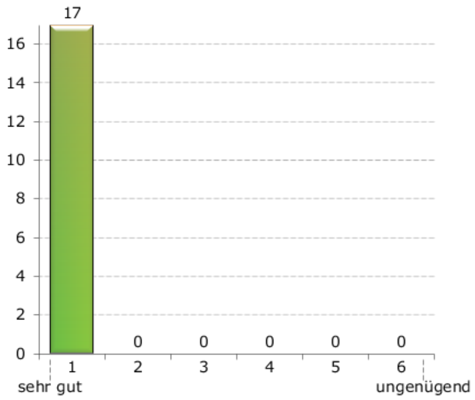


## 1.8 Die Veranstaltung hat pünktlich angefangen.

Antworten: 17 von 22 null 77.3%

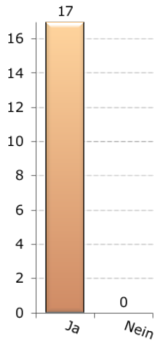


## 1.9 Die Veranstaltung wurde frühzeitig beendet.

Antworten: 16 von 22 null 72.7%

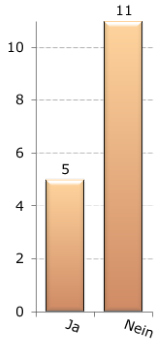


## 1.10 Freie Kommentare :

(sind für die Dozierenden, Studienbeauftragen, Lehrstuhlinhaber und das Studiendekanat einsehbar) Was fanden Sie besonders hilfreich für Ihr Lernergebnis? Was war aus Ihrer Sicht eher hinderlich? Welche konkreten Verbesserungsvorschläge haben Sie?
Antworten: 11 von 22 - Rücklauf: 50.0%

1. Die Veranstaltung schließt insofern sinnvoll an die Lerninhalte anderer Veranstaltungen an, als dass sie eine sinnvolle (und große) Bereicherung der curriculären Inhalte ist und man auch ohne spezifisches Vorwissen in vollem Maße von der Veranstaltung profitieren kann. Man könnte sie aber noch besser ins Curriculum integrieren, indem man ähnliche Veranstaltungen (QB8 bspw.) mit dieser wirklich sehr guten Veranstaltung verzahnt und so der/dem Studierenden einen noch höheren Kompetenzerwerb ermöglicht.

2. Bisher das mit großem Abstand beste Fach im Studium. Organisation und Verzahnung von Theorie und Praxis waren einfach sehr gut. Ich habe keine Antwort bei der Verzahnung zu anderen Veranstaltungen gegeben, da es das so nicht wirklich gibt. Das Thema Katastrophenmedizin und humanitäre Hilfe wird meiner Meinung nach nicht im regulären Studium vermittelt und dennoch ist zumindest die Katastrophenmedizin etwas, was jeder praktisch lernen sollte. Ein großes Dankeschön noch einmal an das ganze Team für diese sehr tolle Woche.

3. Die Veranstaltung fand zu einer guten Uhrzeit statt. Es herrschte eine angenehme Atmosphäre.

4. Ich finde das Wahlfach einmalig und in höchstem Maße innovativ! Aus diesem Grund haben wir als Kurs des WS18/19 Robert Wunderlich auch mit folgendem Text für den Lehrpreis der Universität Tübingen und für den Landeslehrpreis vorgeschlagen und bitten das Dekanat und die Studienkommission diesen Vorschlag zu unterstützen:

   Vielen erscheint es so, als liege der Katastrophenfall für uns in Deutschland in weiter Ferne – diese Gefahren scheinen sich in anderen Teilen dieser Erde abzuspielen, sodass sich die Zivilbevölkerung keine Gedanken darüber macht und auch im Curriculum der Humanmedizin kein verpflichtender Kurs zur „Katastrophenmedizin und Humanitären Hilfe" zu belegen ist. Dank des Engagements von Robert Wunderlich und seinem Team gibt es dennoch ein ganz besonderes Angebot in Form eines 40-stündigen Wahlfachs zu genau diesem Thema. Schon zu Beginn merkt man als Teilnehmer, dass dem gesamten Team um Robert Wunderlich diese Thematik am Herzen liegt und ist von Beginn bis zum Ende gefesselt. Dabei besteht das Team aus einer Vielzahl an Anästhesisten, Psychologen und Ärzten mit jahrzehntelanger Erfahrung im Bereich der Katastrophenmedizin. Zusätzlich arbeitet Robert Wunderlich mit der Feuerwehr zusammen, um eine einmalige Erfahrung für seine Studierenden zu ermöglichen.

   Aber was genau macht das Wahlfach so besonders im Vergleich zu anderen Veranstaltungen, welche die medizinische Fakultät anbietet?
   Diesem Wahlfach geht ein 15-stündiger Online Kurs voraus, welcher die 20 wissbegierigen Studenten, die einen Platz in diesem fantastischen Wahlfach ergattern konnten, auf den eigentlichen 40-stündigen Kurs vorbereitet (Mo-Fr). Dadurch wird eine Basis geschaffen, auf der das Dozententeam perfekt aufbauen kann und wodurch das Wissen in dieser Woche sowohl theoretisch, als auch taktisch und praktisch enorm wachsen kann. Am Ende der Woche hat man dadurch als Student ein gestärktes Selbstbewusstsein und fühlt sich befähigt, sich einem Katastrophenfall stellen zu können. Natürlich sind auch theoretische Einheiten innerhalb des Kurses notwendig, außergewöhnlich ist jedoch, wie diese aufbereitet werden und wie das gesamte Dozententeam und Robert Wunderlich es schaffen, zum selbstständigen Denken und zu interessante Gruppendiskussionen oder Gedankenexperimenten anregen. So zum Beispiel zu ethischen Aspekten der Triage (= nach Behandlungsnotwendigkeit sortieren): Wie entscheidet man sich wer zuerst behandelt wird? Wie bleibt man in Katastrophenfällen professionell und erreicht eine maximale Überlebensrate?
   Darüber hinaus spielen auch humanitäre Fragen eine Rolle, führen zu neuen Betrachtungsweisen und schulen den politischen, gesellschaftlichen und medizinischen Intellekt.

   Zudem sind sehr viele Praxisübungen und mehrere wertvolle Simulationen geboten, wie in noch keinem anderen Kurs des curricularen Medizinstudiums in Tübingen. Beispielsweise eine Computersimulation über das richtige Triagieren – echte Patientenparameter, lebend, schreiend oder auch tot animierte Patienten auf dem PC-Bildschirm, die es zu sichten, zu triagieren und zu versorgen gilt. Außerdem fiktive Szenarien über den Ausruf des Katastrophenfalls in Tübingen, die die Studierenden in Teams (Rettungsleitstelle, Helfer, Krankenhäuser) aufgeteilt bearbeiten. Robert Wunderlich schafft hier mit seinem Team Situationen, die den Studierenden von Beginn an real erscheinen, in welchen unter Stress gearbeitet werden muss und das zuvor Gelernte in die Praxis umgesetzt werden soll. So wissen nun alle Studierenden, was im Falle eines Busunglücks auf der Neckarbrücke, einem Großbrand am Berliner Ring am WHO oder auch bei einem Gasaustritt am Marktplatz logistisch und ressourcentechnisch zu tun ist und wie unter diesen Umständen ein Massenanfall an Verletzten von über 100 Patienten zu versorgen ist.

   Des Weiteren lernt man medizinische Notfallinterventionen, die leider im regulären Medizinstudium zu kurz kommen, aber im täglichen klinischen und notfallmedizinischen Kontext Menschenleben retten können. Diese Skills - beispielsweiße Thorax-Drainagen legen, eine Koniotomie durchführen oder einen intraossären Zugang legen - sollte unserer Meinung nach jede/r Medizinstudent/in und jeder Arzt/Ärztin beherrschen.

   Die Großübung, in welche über 60 Helfer (THW, DRK, JUH, Feuerwehr, viele Ärzte, Psychologen und massig Schauspielpatienten) involviert sind, ist das Herzstück dieses Wahlfachs. Hierbei müssen die Studierenden das Gelernte der gesamten Woche im Team und mit professioneller Kommunikation unter Beweis stellen.

   Zusammenfassend lässt sich sagen, dass wir dieses Wahlfach als ein Privileg ansehen und gerne Robert Wunderlich und das Team um das Projekt mit einem Vorschlag zum Lehrpreis unterstützen möchten. Dadurch erhoffen wir uns, dass noch mehr Studierende von diesem perfekt organisierten und mit Herzblut und viel Engagement entworfenen Wahlfach profitieren können und letztlich Patienten in Notsituationen gerettet und versorgt werden können – und das auch im absoluten Katastrophenfall.

5. Besonders gut war die perfekte Mischung aus lehrreichen Vorträgen und den Praktischen Übungen.
   Dass am Ende sogar noch eine Großünung stattfand war für mich und die anderen Teilnehmer perfekt um nochmals praktisch Erfahrung zu sammeln.
   Rundum eine sehr gute Veranstaltung!

6. Beste Veranstaltung meines gesamten Studiums!! Mehrere sollten die Gelegenheit bekommen an diesem Wahlfach teilzunehmen! Es wurden wichtige Themen besprochen, die auch der ‚normale' Arzt kennen sollte!!

7. Eine der besten Lehrveranstaltungen des Tübinger Studiums.

   Es wäre sinnvoll, wenn die zu evaluierenden Dozenten mit denen im Kurs anwesenden übereinstimmen. Wird das nicht immer nach Abschluss der Lehrveranstaltung beim jeweiligen Lehrbeauftragten nochmals abgefragt?!

8. Die praktischen Teile, sowohl die Planspiele und virtuellen Szenarien, als auch die Realübungen waren super! Man konnte das gelernte einfach direkt anwenden und hatte so auch viele mer das Gefühl effektiv etwas mitgenommen zu haben.

9. Es war die beste Veranstaltung, die ich jemals an der Universität Tübingen besucht habe. Ein außerordentlich motiviertes Team von Dozenten, dass die Balance zwischen Theorie und Praxis perfekt getroffen hat. Ich würde mir für die zukünftigen Studenten wünschen, wenn dieses Wahlfach auch weiterhin angeboten werden kann.

10. Der beste Kurs im ganzen Stuidium. Eine perfekte Mischung aus Wissensvermittlung, praktischer Anwendung und Diskussion. Die Themenwünsche der Kursteilnehmer wurden beachtet und behandelt, so dass keine Frage offen blieb.

11. Ich finde das war einer der besten Kurse des Studiums. Obwohl ich keine Erfahrungen in der Notfallmedizin habe und das auch nicht als späteren Hauptberufswunsch habe, finde ich es essenziel diese Grundlagen erlernt zu haben und ich bin der Meinung dass dieser Kurs in das Curriculum aufgenommen werden sollte.

ostrakon
academia works

# Umfrage Katastrophenmedizin und Humanitäre Hilfe(1)(2)(3)

## Ergebnisse der Umfrage

### Sommersemester 2019

| | | |
|---|---|---|
| Teilnehmer: | 25 | |
| Ausgefüllt: | 24 | (96%) |

Diese Umfrage wurde mit dem Ostrakon Online Evaluation System indicate2 durchgeführt. Bei Fragen die verwendete Technologie betreffend kontaktieren Sie software@ostrakon.org.

# 1 Zur Veranstaltung

## 1.1 Die Lernziele wurden eindeutig definiert.

Antworten: 24 von 25 - Rücklauf: 96.0% - Durchschnitt: 1.12 - Varianz: 0.11 - Standardabweichung: 0.33

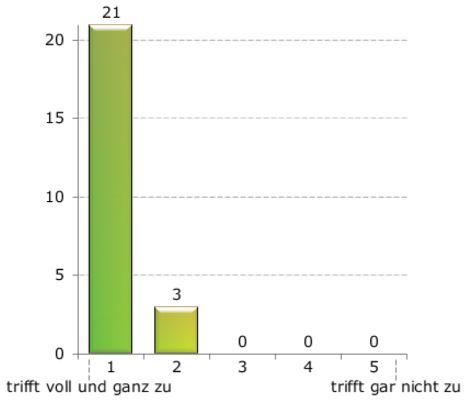


## 1.2 Die Veranstaltung bietet einen guten Überblick über das Themengebiet.

Antworten: 24 von 25 - Rücklauf: 96.0% - Durchschnitt: 1.00 - Varianz: 0.00 - Standardabweichung: 0.00

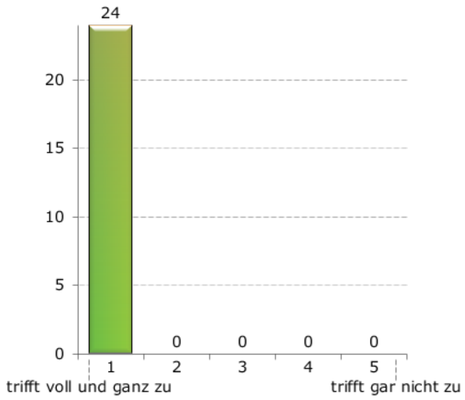


## 1.3 Die Veranstaltung schließt sinnvoll an Lerninhalte anderer Veranstaltungen an.

Antworten: 22 von 25 - Rücklauf: 88.0% - Durchschnitt: 1.50 - Varianz: 0.34 - Standardabweichung: 0.58

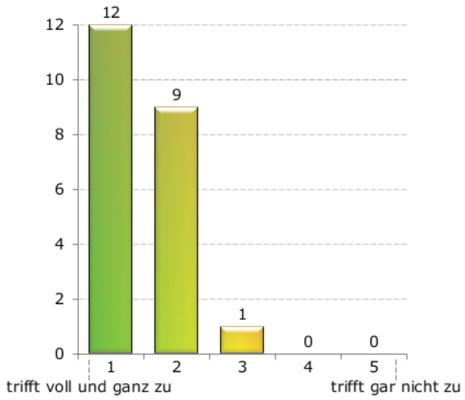

1.4 Die Veranstaltung regt zur Auseinandersetzung mit den behandelten Themen an.

Antworten: 24 von 25 - Rücklauf: 96.0% - Durchschnitt: 1.00 - Varianz: 0.00 - Standardabweichung: 0.00

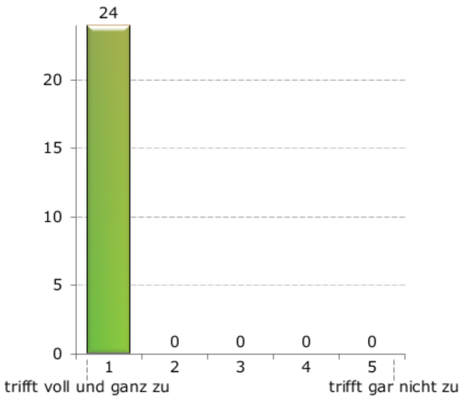


1.5 Die Lehrveranstaltung ist eine gute Mischung aus Wissensvermittlung und Diskussion.

Antworten: 24 von 25 - Rücklauf: 96.0% - Durchschnitt: 1.12 - Varianz: 0.11 - Standardabweichung: 0.33

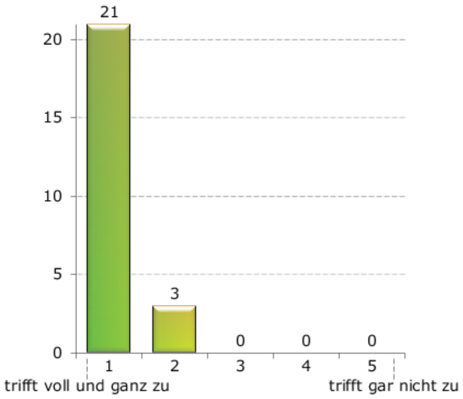


1.6 Mein persönlicher Lernerfolg ist...

Antworten: 24 von 25 - Rücklauf: 96.0% - Durchschnitt: 1.04 - Varianz: 0.04 - Standardabweichung: 0.20

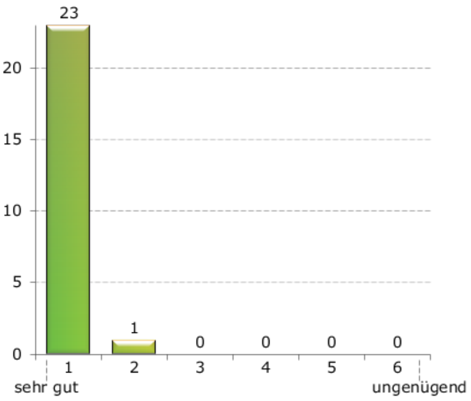

## 1.7 Ich gebe der Veranstaltung die Gesamtbewertung

Antworten: 24 von 25 - Rücklauf: 96.0% - Durchschnitt: 1.00 - Varianz: 0.00 - Standardabweichung: 0.00

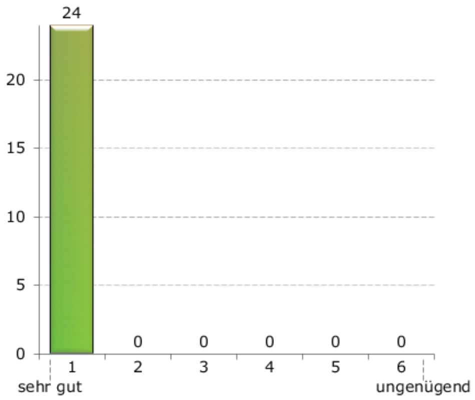


## 1.8 Die Veranstaltung hat pünktlich angefangen.

Antworten: 24 von 25 null 96.0%

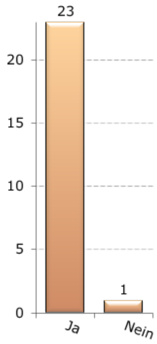


## 1.9 Die Veranstaltung wurde frühzeitig beendet.

Antworten: 22 von 25 null 88.0%

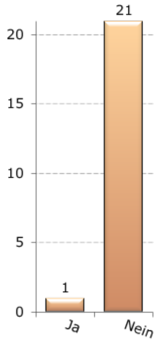


## 1.10 Freie Kommentare :

(sind für die Dozierenden, Studienbeauftragen, Lehrstuhlinhaber und das Studiendekanat einsehbar) Was fanden Sie besonders hilfreich für Ihr Lernergebnis? Was war aus Ihrer Sicht eher hinderlich? Welche konkreten Verbesserungsvorschläge haben Sie?
Antworten: 10 von 25 - Rücklauf: 40.0%

1. Absolut geniales Wahlfach!!

2. Bis dato beste universitäre Veranstaltung! Gute Mischung aus Theorie und Praxis. Kompetente und hochmotivierte Dozenten und Helfer.

3. Einer der besten Kurse bislang, guter Mix aus Vorträgen, Interaktivem und Praktischem. Wegen mir hätte der Kurs auch zwei Wochen mit 80 Stunden gehen können! Nochmal großes Lob an alle!

4. Sehr interessanter Kurs mit vielen Möglichkeiten das gelernte praktisch zu üben.

5. Das war ein tolles Wahlfach, bei dem man auch tatsächlich nachhaltig was gelernt hat. Gute Mischung aus Vortrag und praktischen Übungen.

6. Die beste Lehrveranstaltung der ganzen Klinik. Praxisnah und klinisch relevant!!!

7. Beste Lehrveranstaltung in meiner bisherigen universitären Laufbahn!

8. Bis dato eine der lehrreichsten und didaktisch sinnvollsten Veranstaltungen überhaupt.

9. Die Veranstaltung schließt leider nicht an andere Lehrveranstaltungen an, da humanitäre Hilfe und Katastrophenmedizin bisher nicht im Lehrplan verankert ist.

10. Beste Univeranstaltung, die ich je gehabt habe!!

# Umfrage Katastrophenmedizin und Humanitäre Hilfe(1)(2)(3)(4)

## Ergebnisse der Umfrage

### Wintersemester 19/20

| | | |
|---|---|---|
| Teilnehmer: | 25 | |
| Ausgefüllt: | 25 | (100%) |

Diese Umfrage wurde mit dem Ostrakon Online Evaluation System indicate2 durchgeführt. Bei Fragen die verwendete Technologie betreffend kontaktieren Sie software@ostrakon.org.

# 1 Zur Veranstaltung

## 1.1 Die Lernziele wurden eindeutig definiert.

Antworten: 25 von 25 - Rücklauf: 100.0% - Durchschnitt: 1,20 - Varianz: 0,16 - Standardabweichung: 0,40

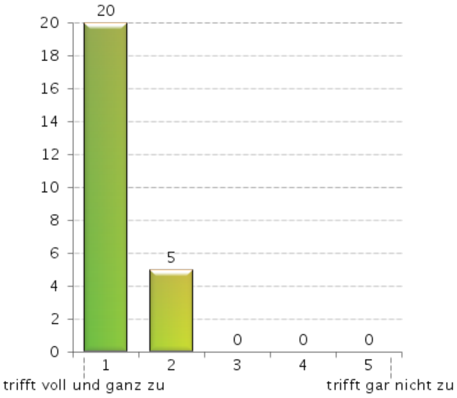


## 1.2 Die Veranstaltung bietet einen guten Überblick über das Themengebiet.

Antworten: 25 von 25 - Rücklauf: 100.0% - Durchschnitt: 1,04 - Varianz: 0,04 - Standardabweichung: 0,20

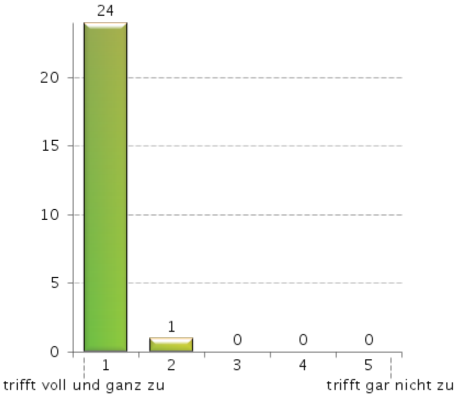


## 1.3 Die Veranstaltung schließt sinnvoll an Lerninhalte anderer Veranstaltungen an.

Antworten: 25 von 25 - Rücklauf: 100.0% - Durchschnitt: 1,36 - Varianz: 0,23 - Standardabweichung: 0,48

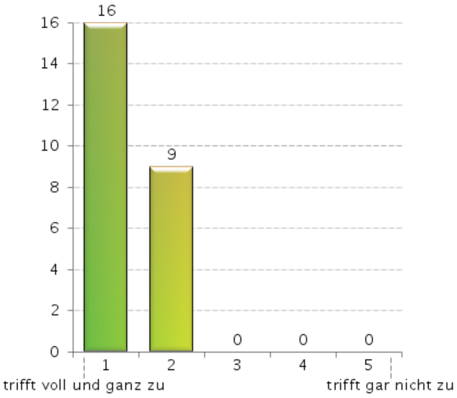

1.4 Die Veranstaltung regt zur Auseinandersetzung mit den behandelten Themen an.

Antworten: 25 von 25 - Rücklauf: 100.0% - Durchschnitt: 1,04 - Varianz: 0,04 - Standardabweichung: 0,20

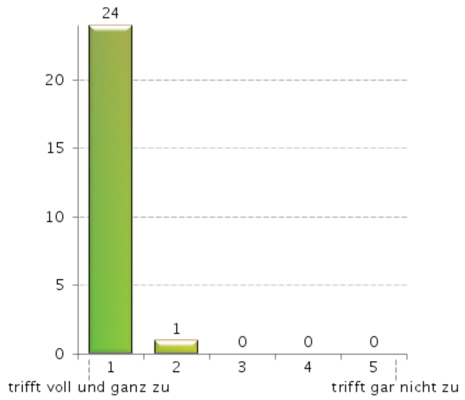


1.5 Die Lehrveranstaltung ist eine gute Mischung aus Wissensvermittlung und Diskussion.

Antworten: 25 von 25 - Rücklauf: 100.0% - Durchschnitt: 1,16 - Varianz: 0,13 - Standardabweichung: 0,37

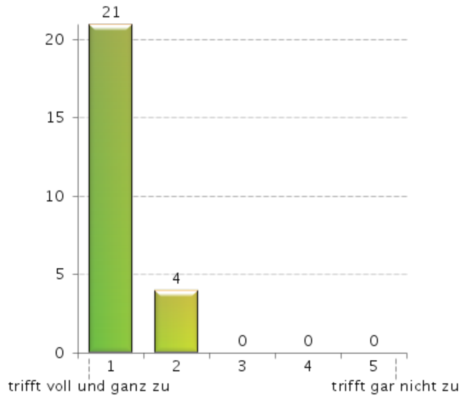


1.6 Mein persönlicher Lernerfolg ist...

Antworten: 25 von 25 - Rücklauf: 100.0% - Durchschnitt: 1,08 - Varianz: 0,07 - Standardabweichung: 0,27

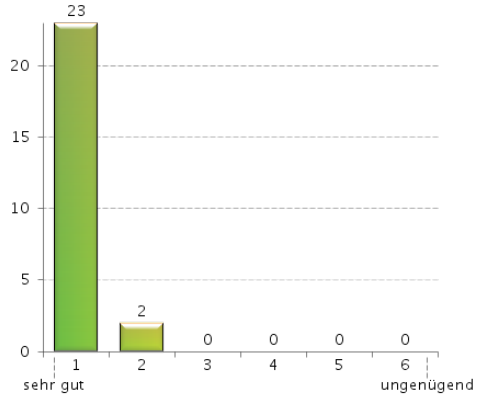

## 1.7 Ich gebe der Veranstaltung die Gesamtbewertung

Antworten: 25 von 25 - Rücklauf: 100.0% - Durchschnitt: 1,04 - Varianz: 0,04 - Standardabweichung: 0,20

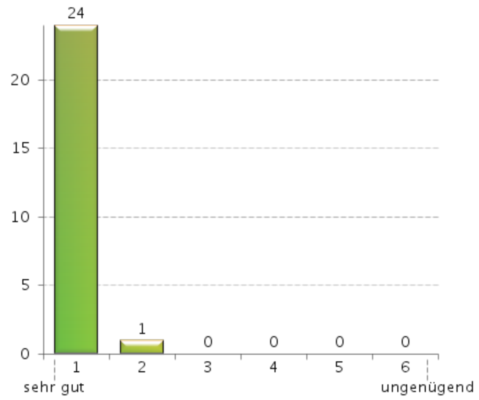


## 1.8 Die Veranstaltung hat pünktlich angefangen.

Antworten: 25 von 25 null 100.0%

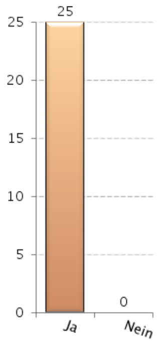


## 1.9 Die Veranstaltung wurde frühzeitig beendet.

Antworten: 24 von 25 null 96.0%

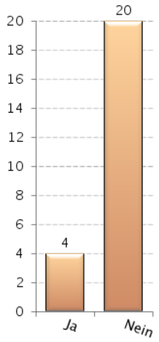


## 1.10 Freie Kommentare :

(sind für die Dozierenden, Studienbeauftragen, Lehrstuhlinhaber und das Studiendekanat einsehbar) Was fanden Sie besonders hilfreich für Ihr Lernergebnis? Was war aus Ihrer Sicht eher hinderlich? Welche konkreten Verbesserungsvorschläge haben Sie?
Antworten: 10 von 25 - Rücklauf: 40.0%

1. das Wahlfach ist sehr toll und sollte curriculär eingeführt werden
vielleicht wäre es gut, noch ein bisschen besser die Aufgaben der jeweiligen Gruppen bei der Großübung zu erklären

2. Beste Veranstaltung die ich je an der Uni hatte

3. Definitiv die beste Veranstaltung die ich an der Uni besucht habe!

4. Super Veranstaltung! Wir bräuchten im Studie mehr Kurse dieser Art (vor allem so interaktive und praxisorientierte Kurse).

5. Dieser Kurs ist der beste Kurs, den ich bisher belegt habe. Die Gestaltung macht das Lernen viel leichter als herkömmliche Veranstaltungen das bisher taten und prägt eine positive, vertraute Atmosphäre für die Dauer des Kurses. Meiner Meinung nach sollte es mehr Kurse geben, die sich an diesem orientieren auch bzw. vor allem im Bereich der Pflichtveranstaltungen.

6. Selten eine so tolle Lehrveranstaltung erlebt. Danke. Am letzten Tag wurden einige wenige Punkte zur Verbesserung mit den Dozenten persönlich besprochen, werden darum hier nicht nochmals aufgeführt.

7. Sehr, sehr motivierte Dozenten, bei denen man merkt, dass sie mit dem Herzen dabei sind. Dadurch auch gute Gruppendynamik. Gute Balance aus Theorie und praktischer Anwendung. Auch die Großübung war eine tolle und im gesamten Studium sicher einzigartige Erfahrung. Vielen Dank!

8. Tolles Lehrformat!

9. Grandiose Veranstaltung. Mehr kann man nicht dazu sagen, keine Kritik, nur positive Eindrücke durchweg durch hochmotivierte Dozenten und eine tolle Lernatmosphäre! Danke danke danke!!!

10. Die gesamte Veranstaltung war sehr gut durchdacht und folgte von Anfang bis Ende einem sinnvollen "roten Faden". Die Organisation war hervorragend und es herrschte ein ausgewogenes Verhältnis zwischen Theorie und Praxis. Die Lerninhalte wurden sehr anschaulich und umfassend dargestellt und die praktischen Übungen schlossen unmittelbar daran an, waren gleichermaßen fordernd und motivierend. Außerdem blieb auch immer Zeit, eigene Fragen einzubringen und zu diskutieren; die eigenständige Arbeit und Mitarbeit wurden aktiv gefördert und ich fühlte mich, auch vonseiten der Dozierenden, sehr gut in das Kursgeschehen integriert. Die Dozierenden haben sehr viel Einsatz und Leidenschaft gezeigt und es so geschafft, uns ihre Begeisterung für ihr Fach zu vermitteln. All diese Faktoren haben maßgeblich dazu beigetragen, einen großen Lernerfolg zu erzielen und nachhaltiges Interesse an der Thematik zu wecken. Insgesamt eine rundum sehr, sehr gelungene Veranstaltung!
